# A solution to the biophysical fractionation of extracellular vesicles: Acoustic Nanoscale Separation via Wave-pillar Excitation Resonance (ANSWER)

**DOI:** 10.1126/sciadv.ade0640

**Published:** 2022-11-23

**Authors:** Jinxin Zhang, Chuyi Chen, Ryan Becker, Joseph Rufo, Shujie Yang, John Mai, Peiran Zhang, Yuyang Gu, Zeyu Wang, Zhehan Ma, Jianping Xia, Nanjing Hao, Zhenhua Tian, David T. W. Wong, Yoel Sadovsky, Luke P. Lee, Tony Jun Huang

**Affiliations:** ^1^ Department of Mechanical Engineering and Materials Science, Duke University, Durham, NC 27708, USA.; ^2^ Department of Biomedical Engineering, Duke University, Durham, NC 27708, USA.; ^3^ Alfred E. Mann Institute for Biomedical Engineering, University of Southern California, Los Angeles, CA 90089, USA.; ^4^ Department of Mechanical Engineering, Virginia Polytechnic Institute and State University, Blacksburg, VA 24060, USA.; ^5^ School of Dentistry and the Departments of Otolaryngology/Head and Neck Surgery, University of California, Los Angeles, Los Angeles, CA 90095, USA.; ^6^ Magee-Womens Research Institute, University of Pittsburgh, Pittsburgh, PA 15213, USA.; ^7^ Department of Obstetrics, Gynecology, and Reproductive Sciences, University of Pittsburgh, Pittsburgh, PA 15261, USA.; ^8^ Renal Division and Division of Engineering in Medicine, Department of Medicine, Brigham and Women’s Hospital, Harvard Medical School, Harvard University, Boston, MA 02115, USA.; ^9^ Department of Bioengineering, Department of Electrical Engineering and Computer Science, University of California, Berkeley, Berkeley, CA 94720, USA.; ^10^ Institute of Quantum Biophysics, Department of Biophysics, Sungkyunkwan University, Suwon, Korea.

## Abstract

High-precision isolation of small extracellular vesicles (sEVs) from biofluids is essential toward developing next-generation liquid biopsies and regenerative therapies. However, current methods of sEV separation require specialized equipment and time-consuming protocols and have difficulties producing highly pure subpopulations of sEVs. Here, we present Acoustic Nanoscale Separation via Wave-pillar Excitation Resonance (ANSWER), which allows single-step, rapid (<10 min), high-purity (>96% small exosomes, >80% exomeres) fractionation of sEV subpopulations from biofluids without the need for any sample preprocessing. Particles are iteratively deflected in a size-selective manner via an excitation resonance. This previously unidentified phenomenon generates patterns of virtual, tunable, pillar-like acoustic field in a fluid using surface acoustic waves. Highly precise sEV fractionation without the need for sample preprocessing or complex nanofabrication methods has been demonstrated using ANSWER, showing potential as a powerful tool that will enable more in-depth studies into the complexity, heterogeneity, and functionality of sEV subpopulations.

## Teaser

sEV subpopulations and nanoparticles are directly fractionated via acoustic virtual wave-pillars without any sample preprocessing.

## INTRODUCTION

Small extracellular vesicles (sEVs) play a crucial role in understanding the systematic pathogenesis of several human diseases (*1*–*7*), including cancer (*8*–*13*), neurodegenerative disease (*14*), and cardiovascular disease (*15*). Currently, there are several commonly used methods to isolate sEVs (extracellular vesicles that are less than 200 nm in diameter) from cell culture media and biofluids (*5*, *16*–*23*), including ultracentrifugation (*24*, *25*), size exclusion chromatography (*26*), polymer-based precipitation (*27*), ultrafiltration (*28*), affinity capture (*29*), magnetic (*30*, *31*), or a combination of the aforementioned methods (*32*). While each method has its own application-specific advantages and disadvantages, they all have limitations preventing a “one-size-fits-all” approach for isolating sEVs. Recently, the use of more precise nanoscale separation technologies, such as asymmetric flow field-flow fractionation (AF4) (*2*), has led to the discovery of distinct sEV subpopulations, which are heterogeneous in size and have distinctive biophysical and molecular characteristics (*12*). These subpopulations of sEVs can broadly be categorized on the basis of their size, with exomeres having diameters smaller than 50 nm, small exosomes (Exo-S) having diameters between 60 and 80 nm, and large exosomes (Exo-L) falling between 90 and 150 nm in diameter. While advances in nanoscale separation technologies have been critical to discovering sEV subpopulations, the need to further elucidate the biogenesis and functionality of sEV subpopulations requires the development of more efficient, fast, and convenient sEV fractionation technologies.

The primary challenge in developing sEV fractionation methods arises from the rapid attenuation of separation forces as the size of the target bioparticles decreases to the nanoscale. This attenuation of nanoscale separation forces, combined with a simultaneous increase in diffusion forces and viscous drag forces (*33*, *34*), makes it challenging to isolate distinct subpopulations of sEVs. Continuous sample processing through force fields or unique nanostructures can help amplify separation forces, enabling high-resolution nanoscale fractionation. AF4 was the first approach used to fractionate sEVs based on their size and hydrodynamic properties, which led to the identification of exomeres. Unlike other sEVs, exomeres are nonmembranous nanoparticles highly enriched in metabolic enzymes and proteins crucial to glycolysis and mammalian target of rapamycin (mTOR) signaling (*2*). sEV fractionation by AF4 also helped reveal the unique protein, lipid, DNA, and RNA profiles of Exo-S and Exo-L. However, AF4 is limited by its requirement for specialized equipment, tedious sample preparation requirements (such as the need first to isolate sEVs via ultracentrifugation), and high sample concentration requirements (*2*, *10*). Nanoscale deterministic lateral displacement (DLD) (*18*) is an alternative nanoparticle separation technology that requires flowing samples through an array of nanometer-sized pillars with carefully chosen gap sizes to deflect nanoparticles in a well-controlled, size-dependent manner. While the iterative separation effect of the nanopillar array enables the sEV separation and fractionation of colloids down to 20 nm, it has extremely low throughputs (~0.1 nl/min) (*18*) and is prone to channel blockage.

**Fig. 1.**
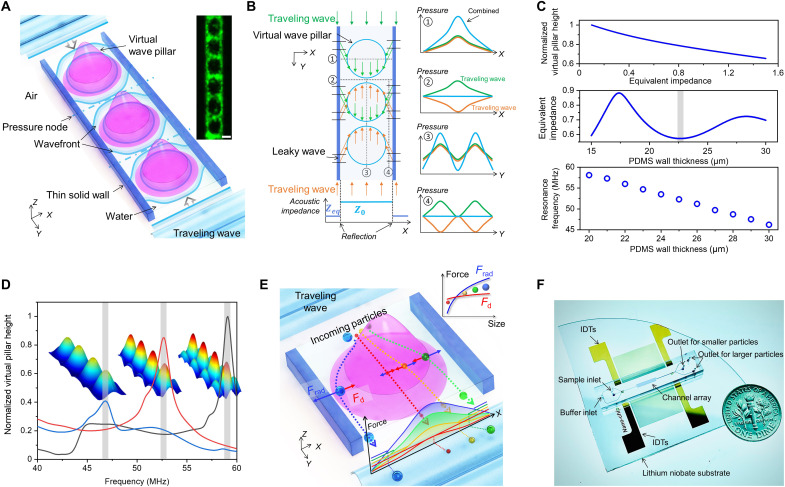
Physical mechanism of ANSWER. (**A**) Schematic showing the generation of acoustic virtual wave pillars via a single pair of IDTs for the biophysical separation of nanoscale bioparticles in a microchannel. Inset, fluorescent polystyrene particles (2 μm) indicate the generation of virtual wave pillars in static flow. Scale bar, 20 μm. (**B**) Wave propagation analysis during the generation of virtual wave pillars. (**C**) Top, calculated normalized virtual pillar height (pressure difference between virtual pillar center and its side) decreases with the increase of external system impedance *Z*_eq_. Middle, external system impedance *Z*_eq_ changes with PDMS sidewall thickness when input frequency is fixed. More detailed results are shown in fig. S3. Bottom, excitation resonance frequency changes with the PDMS sidewall thickness. Gray bars are used to highlight the PDMS wall thicknesses. (**D**) Excitation results in frequency domain under three different conditions. Blue line, channel wall thickness 30 μm; red line, channel wall thickness 25 μm; black line, channel wall thickness 20 μm. Other conditions are identical. Gray bars are used to highlight the resonance frequencies. (**E**) Schematic of nanoparticle separation through acoustic virtual wave-pillar and force analysis. Inset, force analysis for different-sized nanoparticles. *F*_rad_, acoustic radiation force; *F*_d_, drag force. (**F**) Photograph of the ANSWER chip.

In this study, we present a diffraction-based acoustic manipulation strategy, which combines the iterative nature of DLD with tunable, acoustic-based, virtual wave-pillar arrays to achieve a one-size-fits-all separation approach for the fractionation of nanoscale bioparticles (*35*). In our approach, the excitation resonance is used to form tunable acoustic waves in a microfluidic channel, generating an array of virtual acoustic wave pillars (Fig.1, A and B, and figs. S1 and S2). These virtual wave pillars, formed via the overlay of the standing wave field and the orthogonal excitation resonance field, consist of posts (i.e., areas of maximum acoustic pressure or pressure antinodes) and gaps between the posts (i.e., areas of minimum acoustic pressure or pressure nodes). By carefully designing the channel dimensions, we can create a resonance condition where a post is formed at the center of the channel, surrounded by gaps on either side. When particles with a positive acoustic contrast factor flow near the acoustic virtual wave pillars, they experience an excitation resonance–induced acoustic radiation force (*36*–*43*) that pushes them away from the pillar (toward pressure nodes), similar to the deflection of an object against physical pillars. Larger particles experience a more notable excitation resonance–induced radiation force; thus, the virtual wave pillars work as a filter to purify the smaller particles.

On the basis of this mechanism, we developed Acoustic Nanoscale Separation via Wave-pillar Excitation Resonance (ANSWER). ANSWER can be achieved on a single acoustofluidic chip without the need for much of the specialized instrumentation required for DLD or AF4. This offers a simple approach for the separation of sub–100-nm particles with small (<20 nm) diameter differences, a feat that cannot be achieved by current acoustic approaches. Because of its contact-free nature, ANSWER offers a biocompatible approach for the separation of biological nanoparticles. Unlike mechanical filtration methods, which have fixed separation cutoff diameters, ANSWER offers a tunable approach to nanoscale separation and the cutoff diameter can be precisely modified by varying the input acoustic power. Just as the ability to perform high-resolution nanoscale separation of sEVs led to the discovery of distinct subpopulations of sEVs, we believe that our ANSWER platform will expand access to high-resolution nanoscale separation technology and enable previously unidentified discoveries in research areas such as cell biology, molecular biology, biophysics, materials science, drug development, and drug delivery, all of which require the precise separation of nanoscale particles.

## RESULTS

### Physical mechanism of ANSWER

Figure 1 illustrates the physical mechanism of ANSWER. A single pair of interdigital transducers (IDTs) was used to generate a one-dimensional standing surface acoustic wave (SAW) field, which consists of multiple parallel pressure nodes perpendicular to the propagation direction of the SAW. However, when a microfluidic channel with a suitable wall thickness is subjected to this standing SAW field, the surface wave is generated from different points on the IDTs according to Huygens-Fresnel principle and leaks into the liquid from the air through the channel wall (*37*, *44*). When the input frequency is optimized, an excitation resonance in the orthogonal direction of the original wave propagation direction is formed. This excitation resonance is channel-centrosymmetric distributed, with pressure antinodes located at the center of the channel and pressure nodes on either side. This excitation resonance field can be overlaid with the original one-dimensional standing SAW field to form a two-dimensional pillar-shaped antinode distribution we define as virtual wave pillars, as shown in Fig. 1 (A and B). The two necessary conditions to create this phenomenon are sufficient reflection of the leaky waves and the appropriate input frequency.

First, for sufficient reflection, we can divide the microchannel into two parts, the inside liquid and the external system, including the channel wall and adjacent air. Assuming that the equivalent acoustic impedance of this external system is *Z*_eq_, the acoustic impedance of the fluid in the microchannel is represented as *Z*_0_. The reflection coefficient can be written as (*45*)

$$R=\frac{Z_{\mathrm{eq}}-Z_{0}}{Z_{\mathrm{eq}}+Z_{0}}$$ (1)

as *Z*_eq_ is smaller than *Z*_0_, and only considering the absolute value of reflection coefficient, then

$$|R|=|\frac{Z_{\mathrm{eq}}-Z_{0}}{Z_{\mathrm{eq}}+Z_{0}}|=\frac{Z_{0}-Z_{\mathrm{eq}}}{Z_{0}+Z_{\mathrm{eq}}}=1-\frac{2Z_{\mathrm{eq}}}{Z_{0}+Z_{\mathrm{eq}}}$$ (2)

Therefore, to enhance the reflection of the leaky waves (∣*R*∣), the external system impedance should be minimized. Here, we used the virtual pillar height to represent the pressure difference between virtual pillar center and its side and numerically calculated the relationship between the virtual pillar height and the external system impedance *Z*_eq_ when the input frequency is fixed, as shown in Fig. 1C (top), which decreases with an increase of *Z*_eq_. Furthermore, numerical simulations also show that the equivalent impedance *Z*_eq_ is determined by the thickness of the polydimethylsiloxane (PDMS) channel wall when the input frequency is fixed, as shown in Fig. 1C (middle) and fig. S3. The channel wall material used for the calculation was PDMS. Although smaller channel wall thickness can decrease *Z*_eq_ quickly, thin channel walls can increase the risk of liquid leakage from the microchannel in practical application so that local minimum *Z*_eq_ can be considered.

Second, the input frequency should match the resonance frequency of the whole system (*46*, *47*), including the external system described above and the fluid inside the microchannel. This resonance frequency can be obtained by calculating the eigenmodes of the system (fig. S3). As shown in Fig. 1C (bottom), the resonance frequency decreases with the increase of the PDMS sidewall thickness. Figure 1D compares the excitation results in frequency domain under three different conditions. With a PDMS sidewall thickness of 30 μm (blue line), virtual wave pillars with a shallow pillar height are formed at lower resonance frequency. In contrast, a 20-μm sidewall thickness (black line) generates virtual wave pillars with sidelobes at a higher resonance frequency, which decreases the separation efficiency of the system. At a sidewall thickness of 25 μm (red line), virtual wave pillars with a large pillar height can be formed with no sidelobes at their resonance frequency, capable of producing high-efficiency fractionation of nanoparticles.

The fast attenuation of acoustic radiation force and the enhancement of high-frequency SAW-induced acoustic streaming are two limitations restricting current acoustic-based separation down to nanoscale (*48*–*53*). Our ANSWER design can remove the influence of acoustic streaming and improve acoustic radiation force efficiency through the cumulative effect by iterating multiple virtual pillars like DLD technology. Compared to physical pillars, which require highly complex nanofabrication processes and can only be used for one corresponding size, virtual wave pillars eliminate the risk of channel blockage as they can be deactivated. In addition, acoustic virtual wave pillars generated by ANSWER can be electronically tuned to separate nanoparticles across a broad size range, allowing for the selection of various particle cut-off sizes, ranging from 50 to 1000 nm. This is possible because the acoustic radiation force can be adjusted by altering the acoustic pressure distribution. As shown in Fig. 1E, higher acoustic pressure is analogous to a larger virtual wave pillar due to the higher degree of smaller particle deflection. The drag force will counteract this deflection when the virtual wave pillar deflects particles. Although the acoustic radiation force decreases faster than the drag force as the size of the particles decreases, the cut-off size can be adjusted by increasing the acoustic pressure within a specific range, as shown in fig. S4.

Once the system is tuned, larger particles (190 and 100 nm) are almost entirely pushed toward the two side walls as they arrive at the outlet. In this study, we successfully reduced the cut-off size for acoustic-based separation down to 50 nm in continuous flow. We also used ANSWER to isolate different sEV subpopulations directly from human blood plasma without preprocessing the sample.

### Experimental verification of ANSWER

**Fig. 2.**
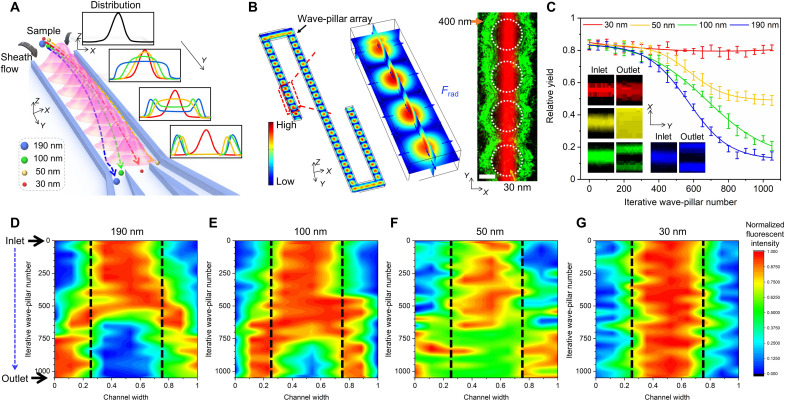
The mechanism and results of nanoparticle separation by iterating multiple virtual wave pillars. (**A**) Schematic showing the iterative process of the accumulation and increase of the minimum lateral deterministic displacement for nanoparticle separation. (**B**) COMSOL simulation (left) showing the acoustic pressure distribution in one channel unit, with a 50-acoustic virtual wave-pillar array. An enlarged fluorescent photomicrograph (right) displays four wave pillars to illustrate the acoustic excitation resonance direction [the direction of the acoustic radiation force, shown in (B) in blue arrows]. Experimental validation of the virtual wave pillars (labeled with white circles) is visualized by imaging a pattern of 400-nm/30-nm polystyrene particles. Scale bar, 10 μm. (**C**) Relative yield (integration of the fluorescence intensity in the collected area divided by integration of fluorescence intensity in all the areas) for the four different size ranges of nanoparticles by the number of wave pillars. Five repeats were used to generate the error bar. Insets show fluorescent images comparing nanoparticle distribution at the channel inlet and outlet in continuous flow. Red, 30 nm; yellow, 50 nm; green, 100 nm; blue, 190 nm polystyrene particles. (**D** to **G**) Normalized fluorescence intensity maps for four sizes [(D) 190 nm; (E) 100 nm; (F) 50 nm; (G) 30 nm] of particles as they travel through the microchannel. The collection regions are labeled by black lines in the center, while waste regions are along the channel sides.

ANSWER uses one pair of IDTs deposited on a lithium niobate (LiNbO_3_) substrate to generate a standing SAW. A PDMS microfluidic channel with multiple parallel units is bonded to the substrate parallel to the direction of SAW propagation (fig. S1). Particles tend to aggregate around the boundary of the wave pillar in static flow but have a tendency to be deflected to the channel sidewalls in continuous flow. At the nanoscale, whether the virtual wave pillar deflects a nanoparticle is dependent on probability. As expected, larger nanoparticles have a higher deflection probability, while smaller particles have a lower deflection probability. Therefore, repeatedly passing particles through multiple virtual wave pillars increases the likelihood of deflection while spatially accumulating and increasing the total lateral deterministic displacement, as shown in Fig. 2A. Our ANSWER chip has 21 repeated serpentine channel sections (Fig. 2B), where each channel section can generate 50 virtual wave pillars, resulting in 1050 wave pillars used to selectively separate nanoparticles. Simulations of the acoustic pressure and force distributions inside the device are shown in Fig. 2B and figs. S5 and S6. When subjected to the applied acoustic profiles, the 400-nm polystyrene particles are distributed near the boundary of the virtual wave pillars (Fig. 2B and movies S1 and S3), verifying the pillar-shaped acoustic pressure distribution. In comparison, the 30-nm polystyrene particles flow uninterrupted through the middle of the channel.

To further investigate the influence of the virtual wave pillar on nanoparticle trajectory, four sizes of nanoparticles (30, 50, 100, and 190 nm) were injected into the microchannel from the center inlet, and sheath flow was injected from the two side inlets. The changes in the fluorescence distribution were recorded for each particle size as they traveled through the channel (figs. S7 to S10). The relative yield was then calculated and shown in Fig. 2C. When they arrived at the outlet, the larger particles (190 and 100 nm) were almost wholly pushed toward the two side walls. The 50-nm particles were partially pushed toward the two walls, while the 30-nm particles remained in the middle of the channel. The inset figures compare the fluorescence distribution at the inlet and outlet for the four different sizes of particles. Figure 2 (D to G) displays the normalized fluorescence intensity maps for each particle size as they traveled through the channel. We observed that the larger particles are deflected further upstream and reach their final lateral migration distance faster than smaller particles. Only a portion of the 50-nm particles was deflected toward the two sides of the channel, and thus, a relatively uniform distribution was achieved at the channel’s outlet. This phenomenon may be explained by the Brownian motion, which works with acoustic radiation force to assist in particle deflection as the concentration gradient decreases from the middle to the sides of the channel (note S1). However, the Brownian motion also works with the drag force to limit deflection when particles reach a uniform distribution. This phenomenon implies that the separation limitation is reached at around 1050 wave pillars. Continuing to increase wave-pillar numbers did not show further improvement on the separation performance.

### Size-selective nanoscale fractionation via ANSWER

**Fig. 3.**
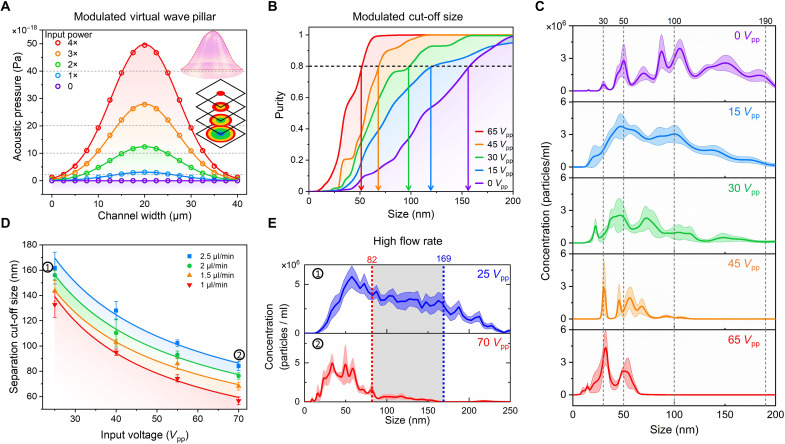
Modulating virtual wave pillars for control of separation cut-off size. (**A**) Numerical computation of the acoustic pressure distribution across the center of the virtual wave pillar (inset) under different input powers. The input power in the simulation was represented by the vibration amplitude of the substrate. Here, 1× input power corresponds to the power required to generate a 0.5-nm vibration amplitude of the substrate. (**B**) Nanoparticle separation cut-off size decreased as input power was increased. A mixture of four different-sized nanoparticles (190, 100, 50, and 30 nm) was used. The purity curves were obtained by integrating the particle size distribution from NTA measurements and divided by the total particle amounts. (**C**) NTA results show the nanoparticle size distributions from collection under different input voltages. (**D**) Comparison of ANSWER’s cut-off size for human plasma separation at different input powers and flow rates. (**E**) At high flow rates (2.5 μl/min for a sample and 3.75 μl/min for sheath flow), the particle cut-off size is reduced from approximately 169 nm (low input power, 25 *V*_pp_) to 82 nm (high input voltage, 70 *V*_pp_), which corresponds to the data points labeled in (D).

We considered two approaches to tune the shape of the virtual wave pillar, namely, frequency modulation and amplitude modulation. The acoustic pressure distribution in one center cross section of a virtual wave pillar was calculated by sweeping at different acoustic frequencies and amplitudes. There was a relatively small effect near the resonant frequency of the IDTs (fig. S11). However, adjusting the amplitude of the input power modulated the acoustic pressure amplitude over a more extensive range (Fig. 3A). This feature enabled the ANSWER device to regulate the acoustic pressure amplitude (e.g., the shape of the virtual wave pillars) by controlling the IDT input power. Here, the conical lines shown in Fig. 3A only represent the acoustic pressure distribution across the channel width, but the actual virtual wave pillar is not a conical shape. Rather, the actual shape of the virtual wave pillar is composed of complex pressure distributions and is presented in detail in fig. S5. We then calculated the purity for the four different sizes of nanoparticles when the input power was increased (fig. S12). These results showed that increasing the input power decreased the separation cut-off size and improved the purity of smaller nanoparticles. A similar experimental result is shown in Fig. 3B. A mixture of the same four sizes of nanoparticles (30, 50, 100, and 190 nm) was injected into the center of the microchannel along with a sheath flow. The size distributions of the collected samples under different input voltages were then measured via the nanoparticle tracking analysis (NTA) system, as shown in Fig. 3C. Purity curves were calculated by integrating particle numbers from 0 to a specific size and dividing by the particle quantity. Corresponding to the simulation results, the purity of smaller nanoparticles increased when a greater input power was deployed, while the purity of larger nanoparticles simultaneously decreased. The cut-off size fell from roughly 157 nm to about 52 nm when the input voltage increased from 0 to 65 *V*_pp_.

Using this phenomenon, we adjusted the cut-off size for blood plasma separation. In Fig. 3D, the flow rate ratio of the blood plasma to sheath flow [phosphate-buffered saline (PBS)] at the inlet was fixed at 1 to 1.5. Four different sample flow rates (1 to 2.5 μl/min) were tested with increasing input voltages applied to the IDTs. All data curves showed the same attenuation trend, ranging from approximately 300 nm to 60 nm. Examples of size distributions for collected bionanoparticles using a flow rate of 2.5 μl/min during minimum and maximum applied acoustic pressures are shown in Fig. 3E. The cut-off size was reduced from 169 nm to 82 nm. Thus, higher flow rates can reduce the time required for particles to travel through the channel and simultaneously increase the drag force, which may cause a higher cut-off size. Compared to the polystyrene nanoparticle separation experiments, the slightly larger cut-off size in the sEV separation experiments may be attributed to a higher viscosity in the plasma, which can increase the drag force. Although Pluronic F-127 (Millipore Sigma, USA) was added to help keep the nanoparticles detached (*54*), the higher surface energy of nanoparticles due to their ultrahigh surface-to-volume ratios and Bjerknes forces from the secondary acoustic radiation force may result in aggregation in a high concentration environment (*55*). However, this effect is much weaker in biofluids such as blood plasma because of its relatively low nanoparticle concentrations, which may also contribute to the result that the cut-off size is slightly smaller in the polystyrene nanoparticle separation experiment than in the sEV separation experiment when a similar input power was applied.

### Fractionation of sEV subpopulations via ANSWER

**Fig. 4.**
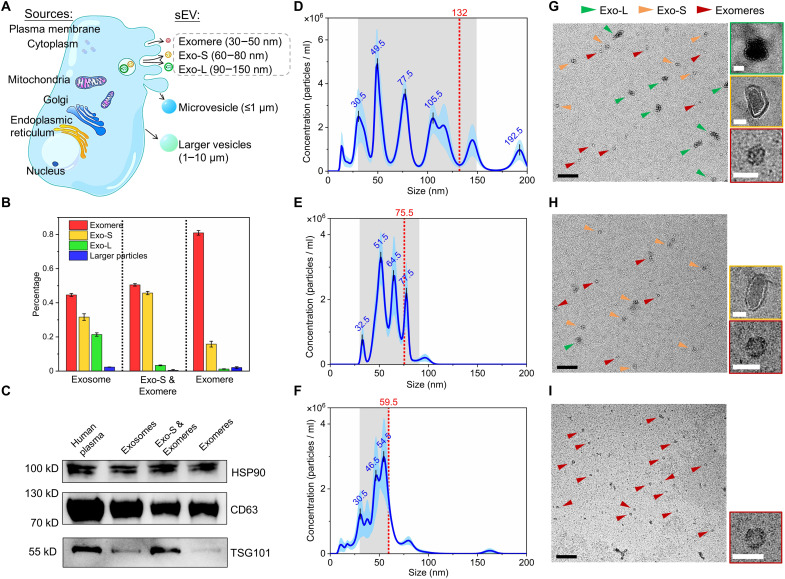
ANSWER performs dynamic isolation of different sEVs directly from human plasma. (**A**) Schematic of different sEV subpopulations generated by cells. (**B**) Calculated proportions of different sEV subpopulations in the isolated samples, including results from (D) to (F). (**C**) Western blot analysis of sEV biomarkers in the isolated samples. (**D**) The particle size distribution in the collected sample, as measured by NTA, shows that sEVs were directly separated from human plasma when low acoustic pressures were applied. (**E**) The particle size distribution in the collected sample, as measured by NTA, shows that only Exo-S and exomere were isolated from human plasma when medium acoustic pressure is applied. (**F**) The particle size distribution in the collected sample, as measured by NTA, shows that only exomere was isolated from human plasma when high acoustic pressure is applied. Each set of NTA data was obtained from at least five NTA assays. Five subsamples were collected and combined for each sample used for NTA to provide measurement. (**G**) TEM image taken from the collected sEV sample. Scale bar, 500 nm. Insets, magnified images of sEV subpopulations with different sizes. Scale bars, 50 nm. (**H**) TEM image taken from collected Exo-S and exomere samples. Scale bar, 500 nm. Insets, magnified images of Exo-S and exomere. Scale bars, 50 nm. (**I**) TEM image is taken from the collected exomere sample. Scale bar, 200 nm. Insets, magnified images of exomere. Scale bars, 50 nm.

ANSWER was deployed to separate sEV subpopulations (Fig. 4A) from the blood via tunable control of the cut-off size. The blood plasma sample was loaded without a preparatory ultracentrifugation step, and pure PBS was used as the sheath flow. The isolated Exo-L and Exo-S samples found sEV biomarkers CD63, TSG101, and HSP90. Only CD63 and HSP90 were found in the isolated exomere sample, suggesting that different sEV subpopulations contain distinct proteins (*2*), as shown in Fig. 4C.

When a relatively low acoustic pressure was applied, sEVs were collected at a purity of approximately 97% (Fig. 4D). Upon increasing the acoustic pressure to a medium level, larger sEVs (Exo-L) were removed from the collection outlet, and the resulting purity of the remaining exomeres and Exo-S was approximately 96% (Fig. 4E). Upon further increasing the acoustic pressure to a relatively high level, the cut-off size was reduced to 59.5 nm, which selectively isolated exomeres with a purity near 80% (Fig. 4F). The different sEV subpopulations isolated by ANSWER were further inspected using transmission electron microscopy (TEM) (Fig. 4, G to I). We found that most sorted vesicles fell within their expected ranges. Figure 4B summarizes these results measured by NTA. We also calculated the proportion of different sEV subpopulations in the collected samples. Specifically, the fractionated sEV subpopulations obtained from the sample shown in Fig. 4F included 81% exomere-size vesicles, 15.8% Exo-S, 1.2% Exo-L, and 2% other larger bioparticles. The statistics of the size distributions calculated from TEM images obtained from these separation results are summarized in fig. S13, which matched the NTA results.

## DISCUSSION

Isolating nanosized objects (<100 nm) is challenging for acoustofluidic devices due to two primary reasons (*56*, *57*). The first is that the acoustic radiation force, typically used for particle separation, rapidly diminishes as the particle size is reduced to the nanoscale. We implemented multiple iterated virtual wave pillars to accumulate and amplify the small difference in forces to separate nanoparticles. The second is the effect of strong acoustic streaming, which can generate strong streaming drag forces that rotate the nanoparticles. However, our ANSWER device design removes the influence of main acoustic streaming. As calculated through COMSOL and verified by our experiment (figs. S5 and S6), particles are mainly streaming in the *y*-*z* plane, which can be observed in the standing SAW field. This streaming cannot be maintained when a continuous flow is implemented in the *y* direction. The flow direction in the channel is orthogonal to the primary streaming axes, which means that the acoustic streaming can only dominate in the static flow. Therefore, acoustic streaming cannot influence the particle’s movement and the separation result. In addition, the influence of the dielectrophoretic effect is excluded by fabricating and testing a similar device with a glass substrate. In this case, no significant difference is observed before and after the radio-frequency signal is applied. IDTs with a long aperture may generate nonuniform acoustic beams (*58*, *59*). However, the excitation resonance mechanism can ensure that the virtual wave pillars are always located in the middle of the microchannel to deflect larger particles to the channel sidewalls.

The relationship between input power and separation cut-off size is summarized in fig. S14. Increasing the input power alone cannot further reduce the cut-off size past 50 nm because of the rapid attenuation of acoustic radiation force. To further reduce the cut-off size, new mechanisms and theories may need to be developed as acoustic radiation force decays in a cubic velocity to the particle size. In addition, developing hybrid nanotweezers by combining acoustofluidic technologies (*60*–*65*) with other nanoscale manipulation technologies, such as plasmonic tweezers (*66*), dielectrophoresis (*67*), and Brownian motors (*33*), may be a promising route toward developing a solution. Furthermore, implementing two-dimensional van der Waals materials holds promise, as they support quasi-particle half-light and half-matter excitations, and exhibit a long lifetime, with low loss and strong field confinement (*68*).

In summary, we have established ANSWER to solve the problem of efficiently separating subpopulations of heterogeneous sEVs. This is particularly germane for the improved understanding of cellular communication pathways and for revealing insights into the role of sEVs in the pathogenesis of various diseases. We showed that ANSWER could be used to obtain high-purity subpopulations of sEVs directly from a biofluid. In addition, we introduced the concept of acoustic virtual wave pillars and have reduced the isolation cut-off size to as low as 50 nm. We believe that ANSWER can play a crucial role in developing sEV-based liquid biopsies by offering a simple, practical, highly accessible approach for sEV fractionation and leading to more quantitative studies of the different molecular content of sEV subpopulations. Our ANSWER technology can be combined with downstream analytical techniques, enabling the analysis of highly pure sEV subpopulations. It can facilitate the development of more advanced tools for the comprehensive understanding of sEVs. This detailed understanding is key to unlocking the diagnostic and therapeutic potential of sEVs in clinical medicine.

## MATERIALS AND METHODS

### Device fabrication and operation

The IDTs were fabricated by depositing a 5-nm-thick layer of Cr followed by a 150-nm-thick layer of Au onto a 128° Y-cut LiNbO_3_ wafer (Precision Micro-Optics, USA) using electron beam evaporation. The photoresist patterns on the LiNbO_3_ wafer used for the metal evaporation were fabricated via photolithography, and the excess metal was removed using a standard lift-off process with acetone. Both IDTs were composed of 40 pairs of electrode fingers. Each finger had a trace width and gap width of 19.5 μm, resulting in an acoustic SAW frequency of 50.5 MHz with an aperture of 16 mm. Using silver epoxy, external wires were bonded to the electrodes (MG Chemicals, USA). The PDMS microchannel was fabricated using standard Su-8 soft lithography and PDMS mold-replica process. A biopsy punch was used to create the inlets and outlets to the microchannel for sample loading and unloading, respectively. The serpentine microchannels consisted of multiple instances of the same base unit. This unit featured a width of 40 μm, a height of 25 μm, and a length of 2.03 mm (vertical main channel) + 0.2 mm (horizontal connection part). These dimensions can ensure that the antinodes are located in the middle of the horizontal sections of the microchannel to facilitate particle separation. When arranged in a serpentine shape, the ANSWER device requires 22 units. The PDMS microchannel and LiNbO_3_ substrate were treated with an oxygen plasma to promote surface bonding, followed by a post-bake at 65°C for 8 hours. A function generator (DG 3012C, Textronix, USA) and an amplifier (25A250A, Amplifier Research, USA) were used to activate the IDTs and generate SAWs. The fluid flow rate and sheath fluid were controlled using syringe pumps (neMESYS, CETONI GmbH, Germany).

### Numerical simulation

Numerical simulation was performed using finite element method (FEM)-based computational modeling software, COMSOL Multiphysics 5.4. Simulations were developed to solve the acoustic field and acoustic streaming distributions in the fluid microchannel. Details can be found in note S2.

### Nanoparticle separation

Polystyrene nanoparticles (30, 50, 100, and 190 nm diameter; Magsphere, USA) were used in the particle separation experiments. Aqueous suspensions (10 μl) of each nanoparticle size were mixed and then diluted into 1 ml of distilled water. Pluronic F-127 (Millipore Sigma, USA) was added to help the nanoparticles remain detached. In the investigation, the flow rates for the nanoparticle mixture and distilled water buffer were set as 1 to 2 μl/min and 1.5 to 3 μl/min, respectively. The frequency used for particle separation was 50.5 MHz, while the voltage applied on the IDTs ranged from 0 to 65 *V*_pp_. The relative yield was calculated by first integrating the fluorescence intensity of the particle in the collection area and then dividing it by the total fluorescence intensity of this kind of particle. The purity was calculated by first counting the number of particles of a specific size range in the collection and dividing it by the total amount of all the collected particles.

### Human plasma separation

The human plasma samples were purchased from Zen-Bio, USA. In the experiment, the flow rate of the plasma sample was set to 1 to 2 μl/min, while the flow rate of the PBS buffer was set to 1.5 to 3 μl/min. The frequency used for separation was 50.5 MHz, and the voltage applied to the IDTs was 25 *V*_pp_ for lower power, 45 *V*_pp_ for medium power, and 70 *V*_pp_ for higher power experiments. A Peltier cooling plate (TEC1-12730, Hebei IT, China) was placed under the ANSWER chip during high power experiments to reduce heating of the chip and to avoid channel blockage. The separation results were verified by a Western blot analysis and TEM imaging.

#### Western blot analysis

The original plasma and isolated products in each experiment (sEVs, small exosome and exomere, exomere) of similar volumes (20 μl) were diluted to 200 μl using PBS. Pierce Cell Lysis Buffer (Thermo Fisher Scientific, USA) and Halt protease inhibitor cocktail (Thermo Fisher Scientific, USA) were used to lyse each sample. After processing by SDS–polyacrylamide gel electrophoresis, the lysates were transferred to a polyvinylidene fluoride membrane (Bio-Rad, USA). This membrane was then incubated with primary antibodies [mouse anti-CD63 (Santa Cruz Biotechnology, USA), mouse anti-HSP90, and rabbit anti-TSG101 (Abcam, UK)] for 12 hours at 4°C, followed by the incubation of appropriate horseradish peroxidase secondary antibody [goat anti-mouse immunoglobulin G (IgG) and goat anti-rabbit IgG (Abcam, UK)] for 1 hour at room temperature. Protein expression levels were finally characterized by ChemiDoc XRS+ (Bio-Rad, USA).

#### TEM imaging

The isolated products from each experiment were processed in the same way. Paraformaldehyde was first added to the sample with a final concentration of 4% (w/v) and then incubated for 20 min at room temperature. A 100-μl droplet of isolated exosome sample was then placed on a sheet of Parafilm (VWR, USA). A 300-mesh copper grid support film (Electron Microscopy Sciences, USA) was placed on the droplet with membrane side down to allow the sample absorption for 20 min. Then, this grid was transferred to a 100-μl droplet of distilled water for 2 min. This process was repeated three times. This grid was then transferred to a 100-μl droplet of uranyl-acetate solution for negative staining for 8 min. Last, the grid was rewashed using distilled water and left to dry at room temperature. The sample was finally observed under TEM (FEI Tecnai G2 Twin, FEI Company, USA).

### Image acquisition and analysis

The images and videos of microscope were acquired using an upright microscope (BX51WI, Olympus, Japan) and a charge-coupled device camera (CoolSNAP HQ2, Photometrics, USA). The data and figures were analyzed using ImageJ (NIH, USA). The nanoparticle size distribution was analyzed using NTA with a NanoSight LM10 apparatus (Amesbury, UK).

## Supplementary Materials

### This PDF file includes:

Notes S1 to S3 Figs. S1 to S14 Table S1 References
