# Supplementary material for: A solution to the biophysical fractionation of extracellular vesicles: Acoustic Nanoscale Separation via Wave-pillar Excitation Resonance (ANSWER)
Source: Sci Adv. 2022 Nov 23;8(47):eade0640. doi: 10.1126/sciadv.ade0640 (PMC9683722; doi:10.1126/sciadv.ade0640)
Supplement: Supplementary file 1 — Notes S1 to S3 Figs. S1 to S14 Table S1 References [file sciadv.ade0640_sm.pdf]

Science Advances
AAAS

Supplementary Materials for

A solution to the biophysical fractionation of extracellular vesicles: Acoustic Nanoscale Separation via Wave-pillar Excitation Resonance (ANSWER)

Jinxin Zhang *et al.*

Corresponding author: Luke P. Lee, lplee@bwh.harvard.edu; Tony Jun Huang, tony.huang@duke.edu

Sci. Adv. **8**, eade0640 (2022)
DOI: 10.1126/sciadv.ade0640

The PDF file includes:

Notes S1 to S3
Figs. S1 to S14
Table S1
Legends for movies S1 to S7
References

Other Supplementary Material for this manuscript includes the following:

Movies S1 to S7

Note S1: Brownian force and Péclet number

The Brownian force applied to a nanoparticle is given by

$$\boldsymbol{F}_B = \zeta \sqrt{\frac{12\pi r_p \mu k_B T}{\Delta t}} \qquad (1)$$

where k_B is the Boltzmann constant; T is the absolute temperature; Δt is the magnitude of the time step; ζ is a Gaussian random number with zero mean and unit standard deviation (*69*). The Brownian force will encourage the particles to spread from high-density regions to the low-density region. The Péclet number, *Pe*, can describe the relative importance of drag force and the Brownian force:

$$Pe = \frac{V_t L}{D} \qquad (2a)$$

$$D = \frac{k_B T}{6\pi \mu r_p}, \qquad V_t = v_{2x} = \frac{3}{8}\frac{{U_1}^2}{c_0}\sin{(4\pi\frac{x}{\lambda})} \qquad (2b)$$

where V_t is the terminal velocity, L is the characteristic length, D is the particle diffusion coefficient, r_p is the particle radius, U_1 is the bulk amplitude, c_0 is the speed of sound in the fluid, λ is the wavelength, x is the displacement (*70*). Here, we set the terminal velocity equal to the maximum streaming velocity (v_{2x}). The characteristic length was set as 10 µm, the spacing between the channel center and collection region boundary. v_{2x} can be calculated on the order of µm/s (*70*). We chose 5 µm/s here. Other parameters and their values are list below:

k_B , the Boltzmann constant: 1.38×10^{-23}

T, the absolute temperature: 293 K (20 °C)

μ, the fluid viscosity: $1.002 \times 10^{-3}\ Ns/m^2$ (water at 20 °C)

For 100 nm polystyrene particles, the diffusion coefficient can be calculated as:

$$D_{100nm} = 4.28 \times 10^{-1}\ \ m^2/s$$

Choosing 10 µm as the characteristic length, the Péclet number can be calculated as:

$$Pe_{100nm} \approx 23.36$$

For 30 nm polystyrene particles, the diffusion coefficient is calculated as:

$$D_{30nm} = 1.43 \times 10^{-1}\ \ m^2/s$$

the corresponding Péclet number is:

$$Pe_{30nm} \approx 7.0$$

Similarly, the Péclet number for other particle sizes can be calculated by changing the size parameter (radius r_p). For sub-100 nm particles, the influence of Brownian force increases when the particle size decreases, but the drag force remains as the dominating force driving the movement of the nanoparticles.

Note S2: Acoustic simulations

Numerical simulations were performed using a FEM-based computational modeling software package, COMSOL Multiphysics 5.4, to solve the acoustic field distributions and acoustic streaming present in the fluid channel and analyze the nanoparticle movement under the influence of the acoustic radiation force (*55-57*).

The flow motion in the channel is governed by the Navier-Stokes equations:

$$\frac{\partial \rho}{\partial t} + \nabla \cdot (\rho \boldsymbol{v}) = 0 \tag{3}$$

$$\rho \frac{\partial \boldsymbol{v}}{\partial t} + \rho(\boldsymbol{v} \cdot \nabla) = -\nabla p + \mu \nabla^2 \boldsymbol{v} + \left(\mu_b + \frac{1}{3}\mu\right) \nabla(\nabla \cdot \boldsymbol{v}) \tag{4}$$

where ρ, μ, and μ_B represent the density, dynamic viscosity, and bulk viscosity of fluid, respectively; p and $\boldsymbol{v}$ are the fluid velocity and pressure, respectively. By applying perturbation theory, the 1st order equations governing the acoustic field and 2nd order equations governing acoustic streaming can be deduced as (*66*):

$$i\omega\rho_1 + \rho_0 \nabla \cdot (\boldsymbol{v_1}) = 0 \tag{5}$$

$$\rho_0 i\omega \boldsymbol{v_1} = -c_0^2 \nabla \rho_1 + \mu \nabla^2 \boldsymbol{v_1} + \left(\frac{1}{3}\mu + \mu_B\right) \nabla(\nabla \cdot \boldsymbol{v_1}) \tag{6}$$

$$\rho_0 \nabla \cdot \langle \boldsymbol{v_2} \rangle = -\nabla \cdot \langle \rho_1 \boldsymbol{v_1} \rangle \tag{7}$$

$$-\nabla\langle p_2 \rangle + \mu \nabla^2 \langle \boldsymbol{v_2} \rangle + \left(\frac{1}{3}\mu + \mu_B\right) \nabla(\nabla \cdot \langle \boldsymbol{v_2} \rangle) = \rho_0 \langle j\omega \boldsymbol{v_1} \rangle + \rho_0 \langle (\boldsymbol{v_1} \cdot \nabla) \boldsymbol{v_1} \rangle \tag{8}$$

In the 1st order equations (equation (5) and (6)), c_0,ρ_0, μ, and μ_B are the speed of sound in the fluid, the density, shear viscosity, and bulk viscosity of fluid, respectively; ρ_1 and $\boldsymbol{v_1}$ are the first order density and velocity. The acoustic pressure can be obtained using $p_1 = c_0^2 \rho_1$. In the 2nd order equations (equation (7) and (8)), the brackets $\langle \cdot \rangle$ indicates a time average over a harmonic oscillation period; p_2 is the second order pressure and $\boldsymbol{v_2}$ is the second order velocity. The time-averaged second order velocity is physically the acoustic streaming velocity. In our device, the vibration velocity of the channel floor induced by the standing SAW propagates in the y-direction and can be expressed as

$$v_y = \varepsilon A\omega\left\{e^{i(\omega t - k\ ) - \alpha y} + e^{i[\omega t - k(y_0 - y) - \alpha(y_0 - y)]}\right\} \tag{9}$$

$$v_z = -iA\omega\left\{e^{i(\omega t - ky) - \alpha y} - e^{i[\omega t - k(y_0 - y) - \alpha(y_0 - y)]}\right\} \tag{10}$$

where ε is the ratio between longitudinal and transverse vibrations, A is the transverse vibration amplitude of the substrate, α is the decay coefficient of the vibration along the direction of the wave propagation, k is the wave number of the standing SAW, and y_0 is the width of the fluid domain in y direction. In the COMSOL simulation package, the "Thermoviscous Acoustics" physics module was applied to solve the 1st order equations. Equation (9) and (10) were applied as the velocity at the bottom of the fluid, as the boundary conditions. The impedance of the PDMS was applied to the sides and top of the channel using the "Impedance" boundary condition. The model was solved using a "Frequency Domain" solver at the resonant frequency of the device. The "Laminar Flow" physics model was applied to solve the 2nd order equations and solved for a

“Stationary” case. The “No slip” wall condition is applied to all the channel walls. More details of this simulation model can be found here (*71*).

Note S3: Input power evaluation

The input power can be evaluated as

$$P = \frac{{V_{rms}}^2}{Z}$$

Here V_{rms} is the RMS voltage in AC signal, and Z is the impedance. V_{rms} is related to the input voltage V_{pp}, as shown below,

$$V_{rms} = \frac{V_{peak}}{\sqrt{2}} = \frac{V_{pp}/2}{\sqrt{2}} = \frac{V_{pp}}{2\sqrt{2}}$$

Therefore, the input power can be written as:

$$P = \frac{{V_{rms}}^2}{Z} = \frac{{V_{pp}}^2}{8Z}$$

The impedance can be measured and then the input power can be evaluated. Here, the impedance of our device is around 450Ω.

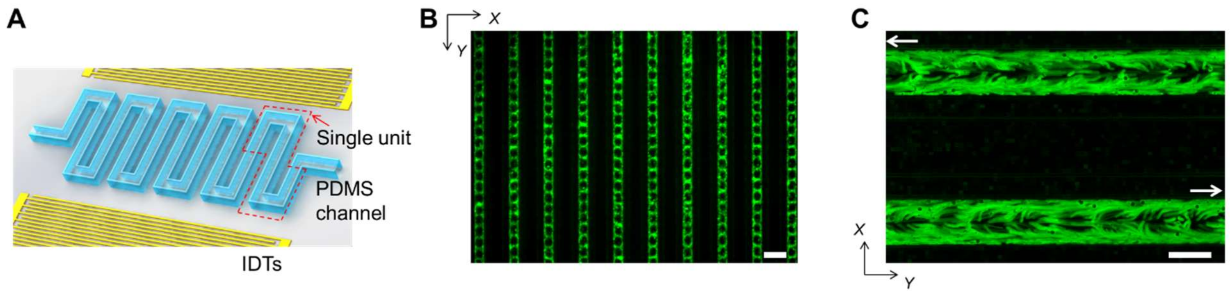

Fig. S1. ANSWER device design. (A) Schematic of the ANSWER chip layout. **(B)** The original image from which the inset of Fig. 1a was obtained, showing the virtual wave-pillar distribution. Scale bar, 100 μm. **(C)** Time-lapse stacked images showing that 2 μm polystyrene particles are pushed to the two sides of the fluid microchannel due to wave-pillar excitation resonance. The white arrows indicate the flow direction. Scale bar, 40 μm.

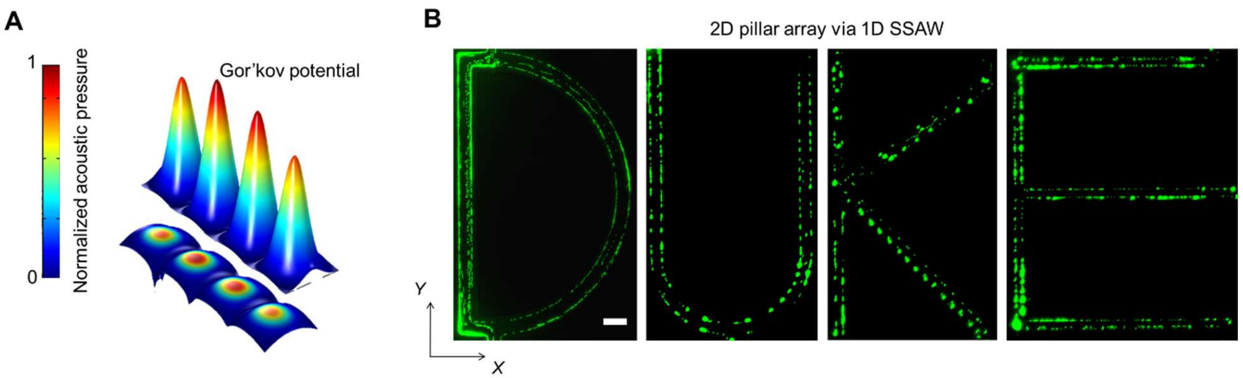

Fig. S2. Two-dimensional virtual wave-pillar generation via one pair of IDTs in ANSWER. **(A)** Numerical calculations show the Gor'kov potential (acoustic pressure) distribution in a pillar shape. **(B)** Microscope images showing 2 µm polystyrene particle patterns in a "DUKE" shaped channel with different widths ranging from 60 µm to 30 µm under the channel aligned SAW. Because of the interaction between the acoustic waves and the thin PDMS channel, surface acoustic waves can be aligned to generate pressure nodes close to the channel walls even if they are not straight. Scale bar: 100 µm. Channel width for "D", "U", "K", "E" is 60 µm, 50 µm, 40 µm, and 30 µm, respectively.

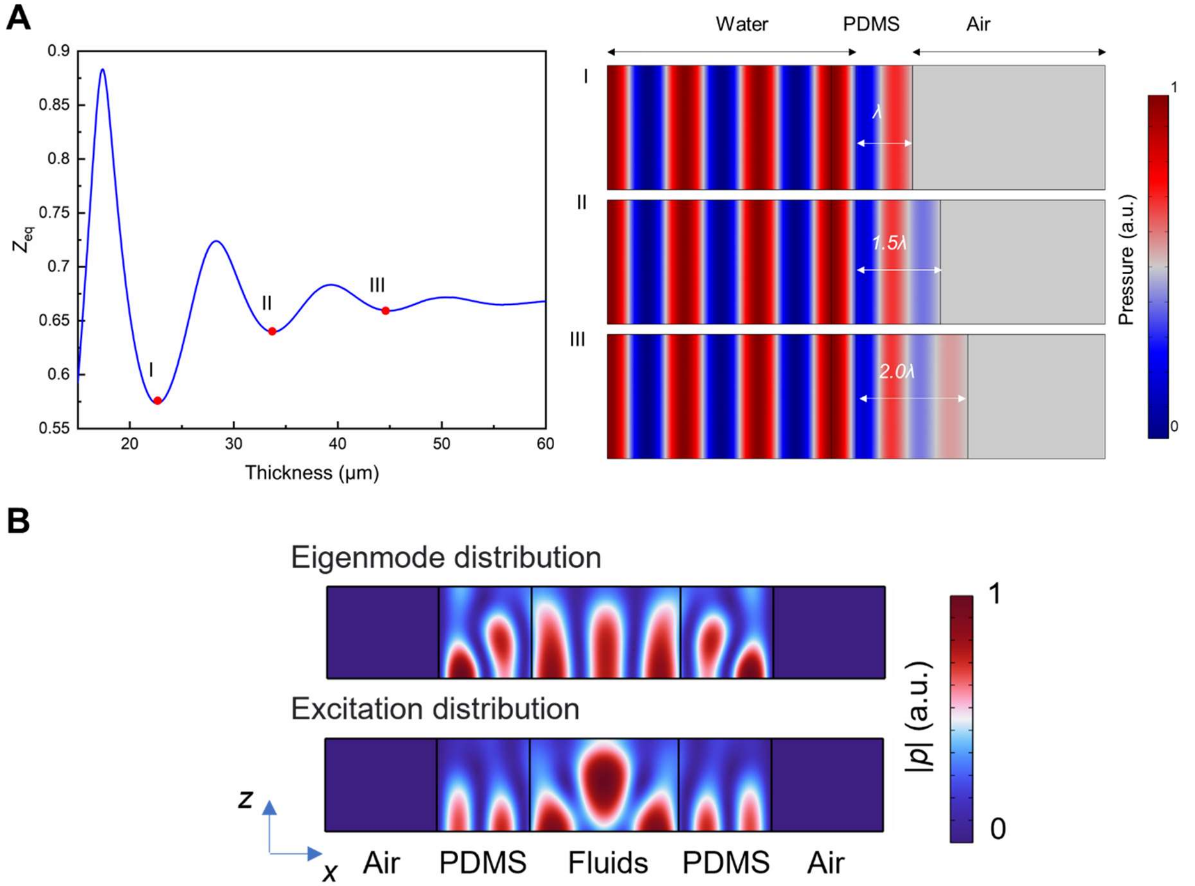

Fig. S3. Numerical calculation for excitation resonance effect. (A) Numerical simulations showing the relationship between equivalent impedance and the thickness of the PDMS wall. The equivalent impedance is influenced by the resonance of the PDMS. The equivalent impedance Z_{eq} was calculated for increasing PDMS thicknesses in a wider range (from 15 μm to 60 μm). The first valley (I) corresponds to a resonance mode when the PDMS thickness is closest to one wavelength (λ). When the thickness increases to 1.5λ and 2.0λ, the Z_{eq} has second (II) and third (III) resonance modes. Therefore, when the PDMS wall thicknesses are in integer multiples of half wavelength, the resonance modes will result in a local minimum value. The Z_{eq} will increase and drop between two resonance modes. **(B)** Simulation of the excitation resonance frequency of the ANSWER system. The resonance frequency is obtained by calculating the eigenmodes of the system.

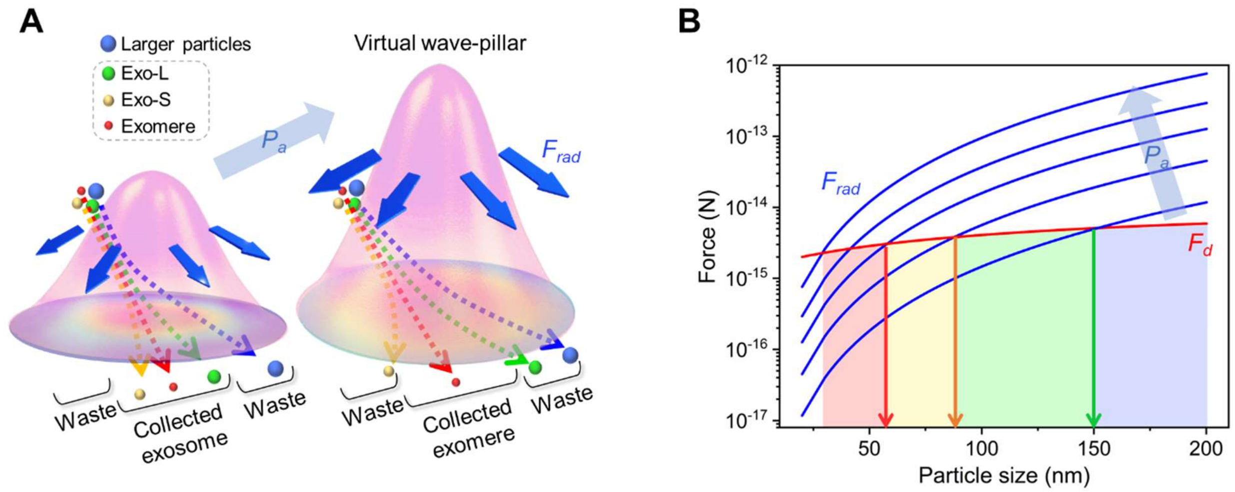

Fig. S4. Modulating the virtual wave-pillar to control the cut-off size. (A) Illustration of the modulated virtual wave-pillar under increasing acoustic pressure used for separating different sEV subpopulations. P_a: acoustic pressure. **(B)** Numerical results showing dynamic control of the separation cut-off size, achieved by adjusting the acoustic pressure.

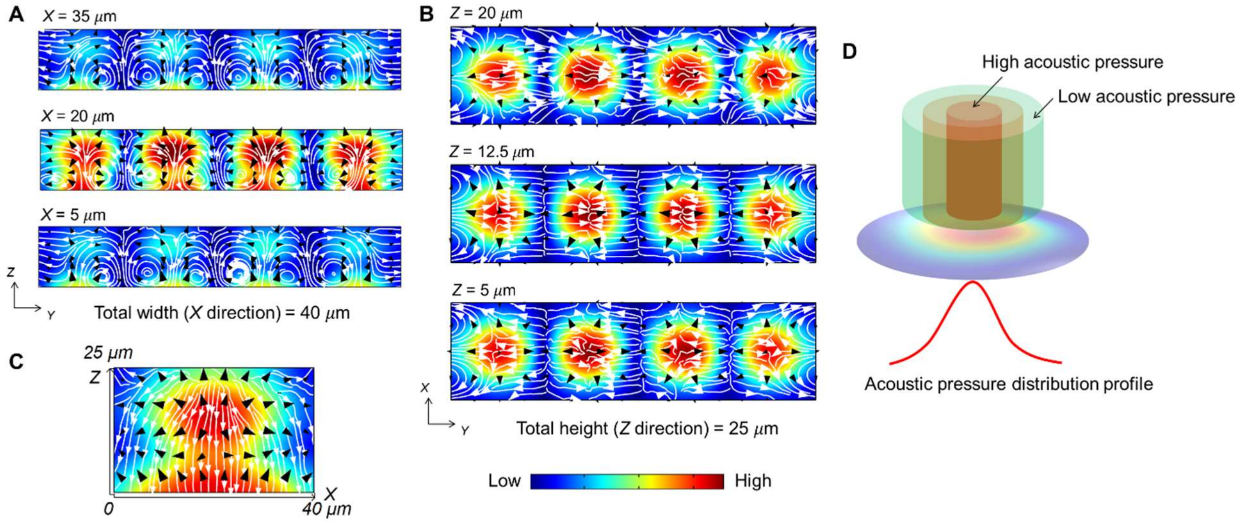

Fig. S5. Numerical simulation results showing the acoustic pressure distribution and streaming in different channel planes. (**A)** In the *z-y* plane, acoustic pressure and streamline distribution at different *x* slices. Channel width is 40 μm, $0 \le x \le 40$ μm. (**B)** In the *x-y* plane, acoustic pressure and streamline distribution at different *z* slices. Channel height is 25 μm, $0 \le z \le 25$ μm. (**C)** In the *z-x* plane, acoustic pressure and streamline distribution at one virtual wave-pillar center's cross-section. The white lines are streamlines. The black arrows point the direction of acoustic radiation force. (**D**) The acoustic pressure distribution inside the channel most closely resembles a series of concentric cylinders, although the actual shape of the acoustic pressure distribution is far more complex, as shown in (**A-C**).

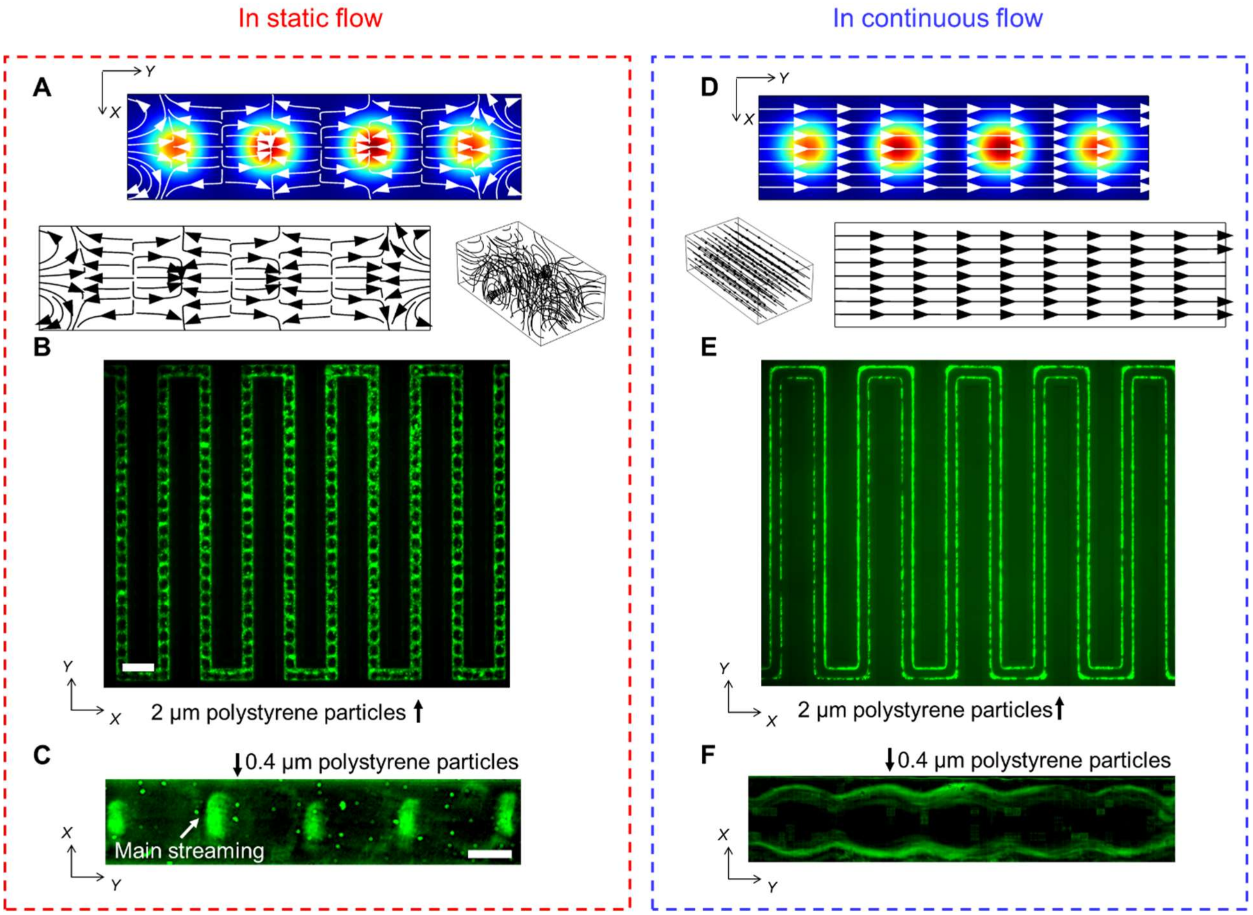

Fig. S6. Comparison of the acoustic patterns and acoustic streaming effects in static flow and continuous flow. In static flow (*i.e.*, the flow rate is zero): **(A)** streamline distribution when acoustic pressure is applied, the streaming is observed in the *y* direction. (**B)** 2 µm and **(C)** 400 nm polystyrene particle distribution when the acoustic field is applied. The movement of 2 µm particles is dominated by the acoustic radiation force, while the movement of 400 nm particles is dominated by the acoustic streaming. These experimental results are in good agreement with the simulation results. In continuous flow: **(D)** streamline distribution when acoustic pressure is applied, the effect of streaming is removed from the flow in the *y* direction. **(E)** 2 µm and **(F)** 400 nm polystyrene particles distribution when acoustic pressure is applied. In continuous flow, 400 nm particles can be pushed to the two sides *via* acoustic radiation force due to the absence of acoustic streaming. Scale bar in (B) and (E), 100 µm. Scale bar in (C) and (F), 20 µm.

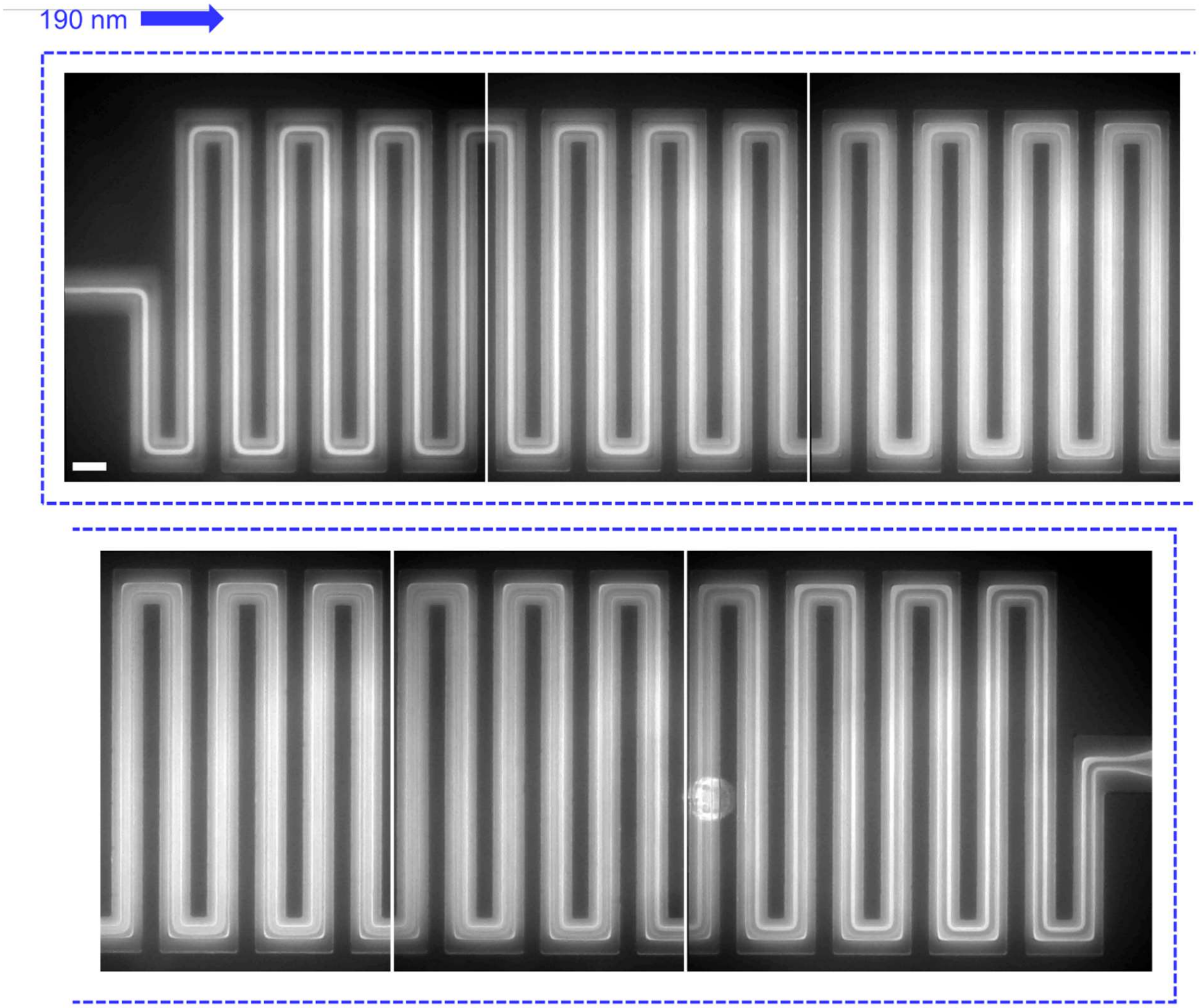

Fig. S7. Photomicrographs showing the change in 190 nm polystyrene particle distribution as the particles are iteratively separated through the ANSWER device. The 190 nm polystyrene particles were loaded from the center of the channel inlet while sheath flow was loaded from two side inlets. From upper left to the lower right, the particles iterate through around 1050 virtual wave-pillars. As they flow through the chip, the 190 nm particles are moved to two sides at the outlet. Scale bar, 100 µm.

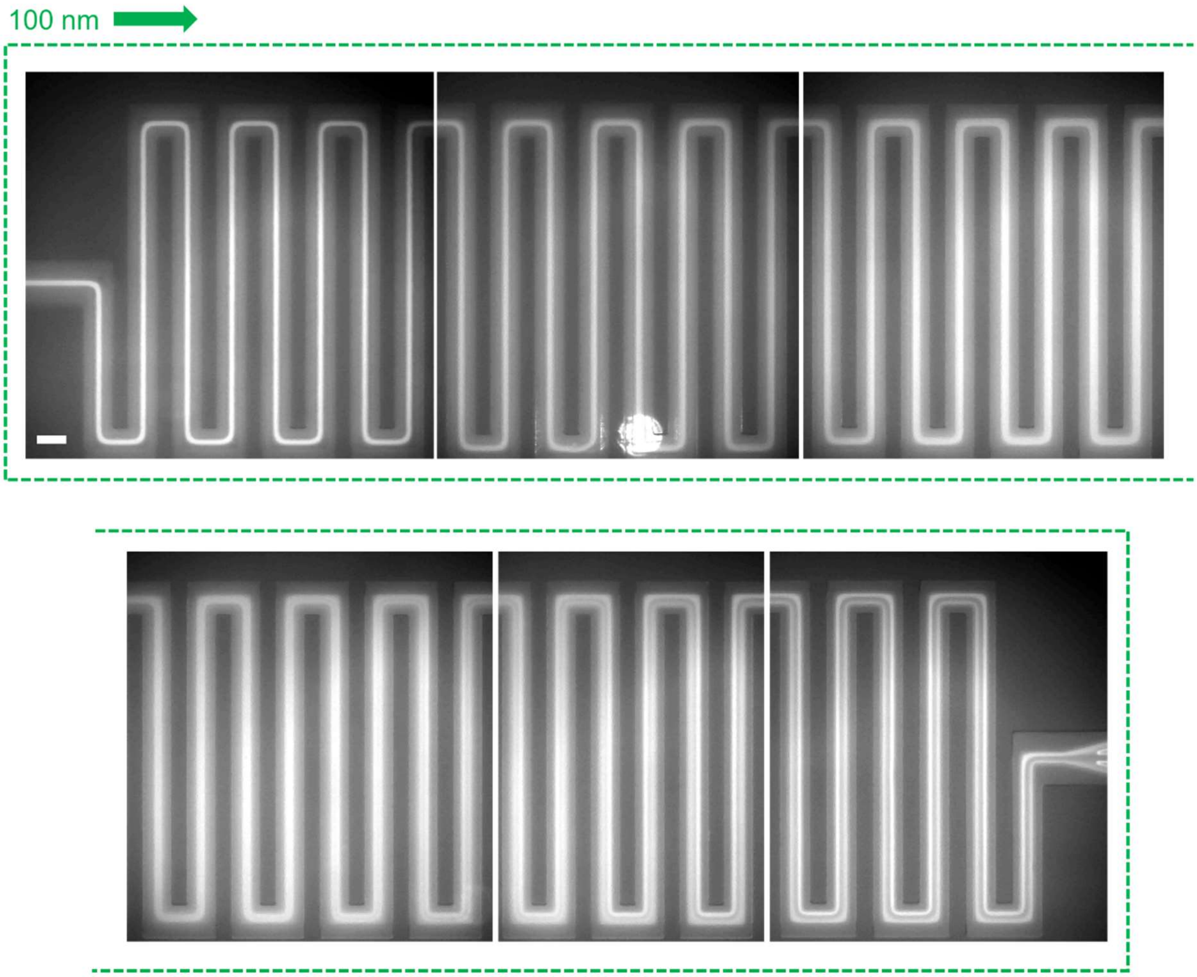

Fig. S8. Photomicrographs showing the changes in 100 nm polystyrene particle distribution as the particles are iteratively separated through the ANSWER device. The 100 nm polystyrene particles were loaded from the center of the channel inlet while sheath flow was loaded from two side inlets. From upper left to the lower right, the particles iterate through around 1050 virtual wave-pillars. As they flow through the chip, the 190 nm particles are moved to two sides at the outlet. Scale bar, 100 µm.

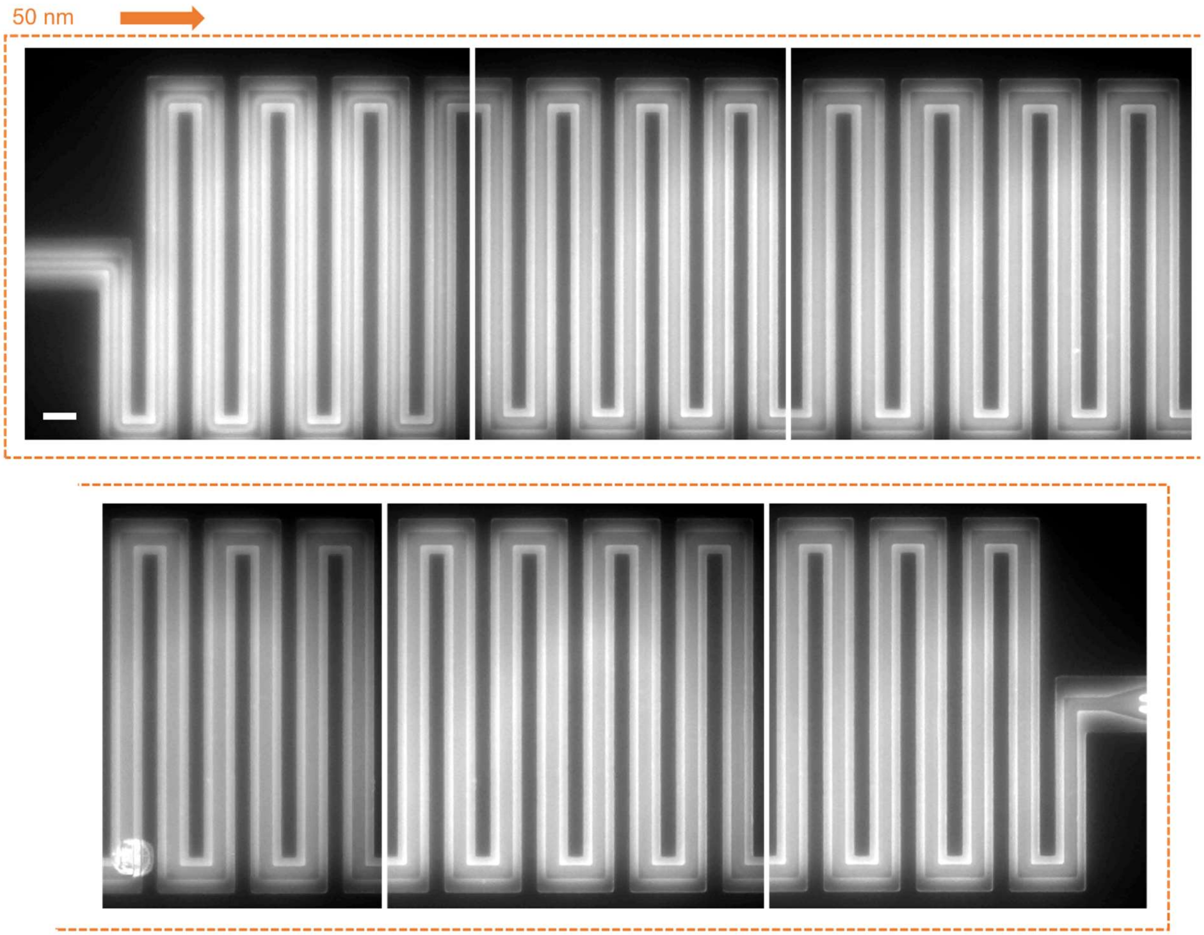

Fig. S9. Photomicrographs showing the changes in 50 nm polystyrene particle distribution as the particles are iteratively separated through the ANSWER device. The 50 nm polystyrene particles were loaded from the center of the channel inlet while sheath flow was loaded from two side inlets. From upper left to the lower right, the particles iterate through around 1050 virtual wave-pillars. As they flow through the chip, the 50 nm particles are moved to two sides at the outlet. Scale bar, 100 µm.

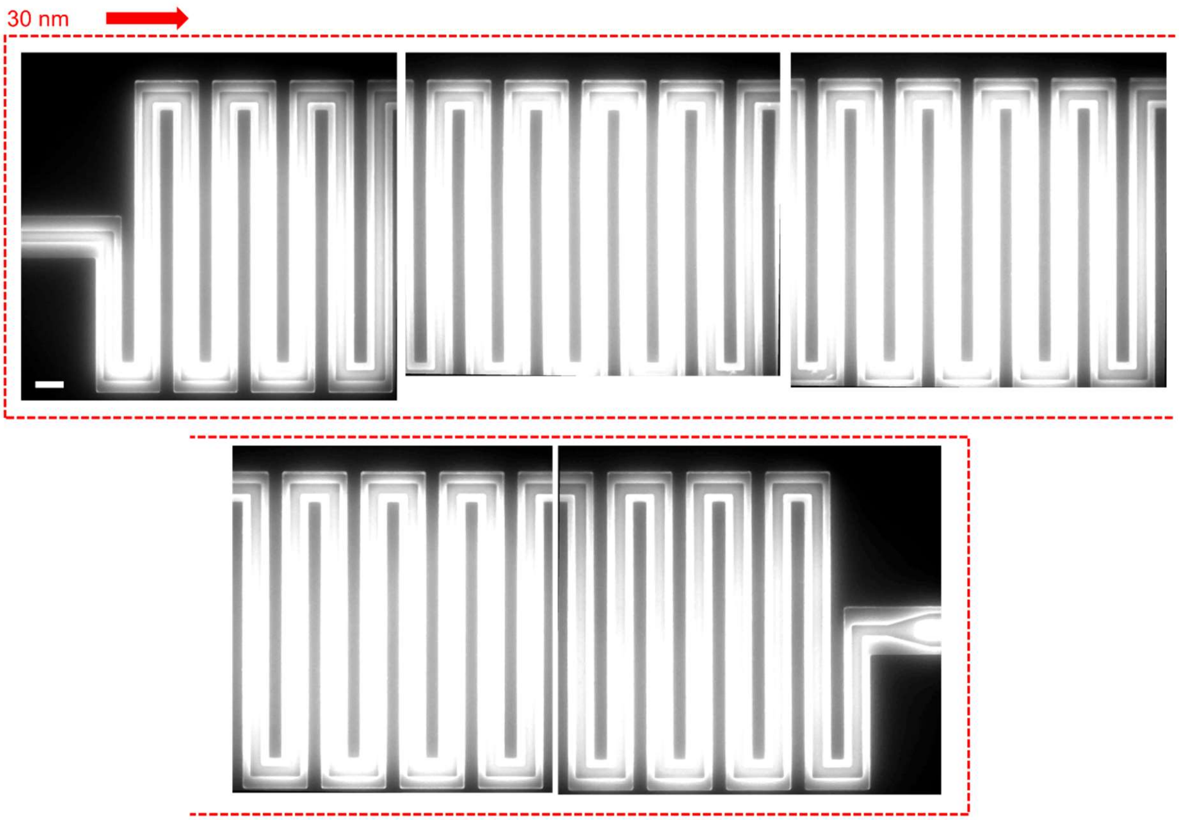

Fig. S10. Photomicrographs showing the changes in 30 nm polystyrene particle distribution as the particles are iteratively separated through the ANSWER device. The 30 nm polystyrene particles were loaded from the center of the channel inlet while sheath flow was loaded from two side inlets. From upper left to the lower right, the particles iterate through around 1050 virtual wave-pillars. The particles were always in the middle of the channel and did not get pushed to the channel sidewalls. Scale bar, 100 µm.

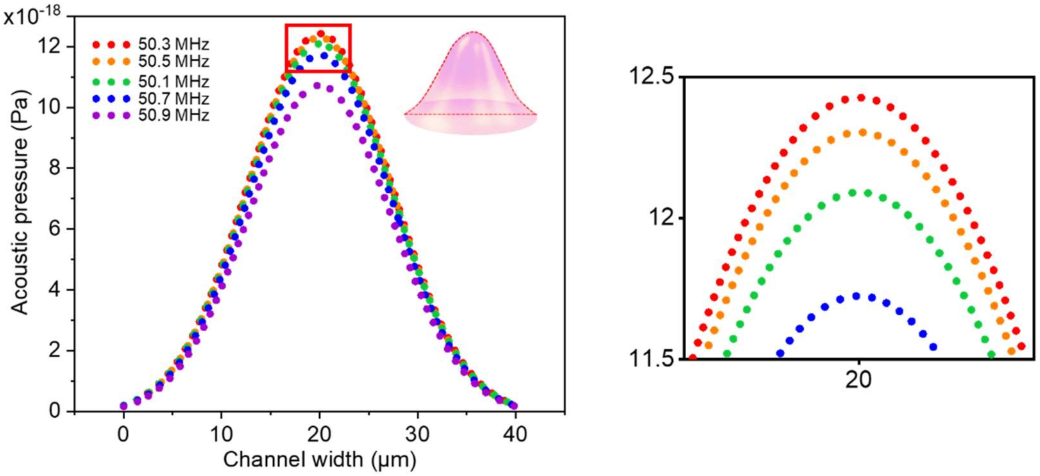

Fig. S11. Modulated wave-pillar amplitude *via* frequency tuning. Numerical computation of the acoustic pressure distribution across the center cross-section of the virtual wave-pillar (inset) under different frequencies centered around the resonance frequency. The right figure shows a zoomed-in view of the virtual pillar pressure distribution (left red box).

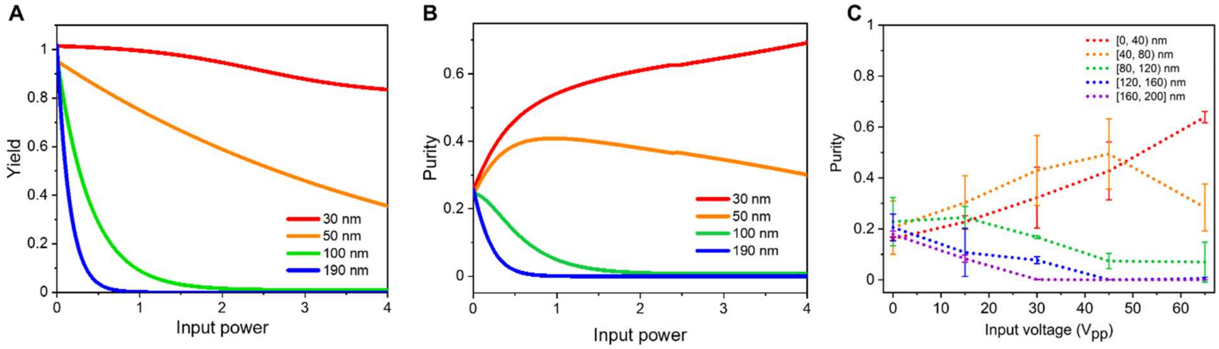

Fig. S12. Nanoparticle separation quality as a function of input power. Numerically calculated **(A)** yield and **(B)** purity for four different sized particles changes with increased input power. $Yield = \frac{collected\ particles\ with\ this\ size}{all\ the\ particles\ with\ this\ size}$, $Purity = \frac{collected\ particles\ with\ this\ size}{all\ the\ collected\ particles}$. The input power is characterized by the substrate vibration amplitude. Specifically, 1× input power is defined as the power required to generate a substrate vibration amplitude of 0.5 nm. **(C)** Experimental verification of selectively isolating nanoparticles *via* adjusting the input power. The purity of different nanoparticle size ranges changes with increased input voltage.

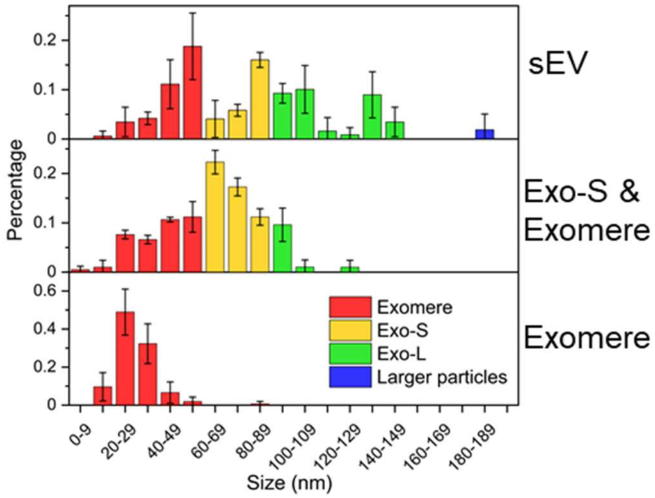

Fig. S13. Size distributions collected from TEM images obtained from three separation results.

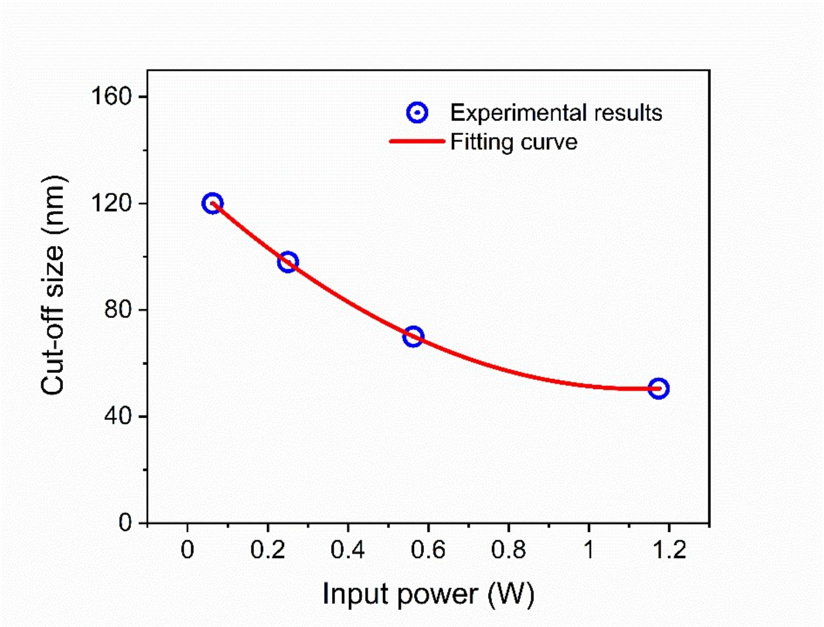

Fig. S14. The relationship between input power and nanoparticle separation cut-off size.

Table S1. Parameters used in numerical simulation.

Parameter	Symbol	Value
Density of the water	ρ_{water}	$997\ kg/m^3$
Density of the PBS	ρ_{PBS}	$1040\ kg/m^3$
Shear viscosity of the water	μ_{water}	$1 \times 10^{-3}\ Pa \cdot s$
Shear viscosity of the PBS	μ_{PBS}	$0.9 \times 10^{-3}\ Pa \cdot s$
Amplitude of substrate vibration	A_m	0, 0.5, 1, 1.5, 2 nm
Frequency of the SSAW	f	50.1, 50.3, 50.5, 50.7 50.9 MHz
Density of polystyrene particles	ρ_P	$1050\ kg/m^3$
Diameter of polystyrene particles	d_P	30, 50, 100, 190 nm

Movie S1: Visualization of acoustic virtual wave-pillars *via* 2 μm polystyrene particle patterns in the continuous flow.

Movie S2: Visualization of acoustic streaming influence *via* 0.4 μm polystyrene particle patterns in the static flow.

Movie S3: Removal of the acoustic streaming effect in continuous flow verified via 0.4 μm polystyrene particle patterns.

Movie S4: 190 nm polystyrene particle distribution changes as they iterate through the ANSWER device.

Movie S5: 100 nm polystyrene particle distribution changes as they iterate through the ANSWER device.

Movie S6: 50 nm polystyrene particle distribution changes as they iterate through the ANSWER device.

Movie S7: 30 nm polystyrene particle distribution changes as they iterate through the ANSWER device.

REFERENCES AND NOTES

1. R. Kalluri, V. S. LeBleu, The biology, function, and biomedical applications of exosomes. *Science* **367**, eaau6977 (2020).

2. H. Zhang, D. Freitas, H. S. Kim, K. Fabijanic, Z. Li, H. Chen, M. T. Mark, H. Molina, A. B. Martin, L. Bojmar, J. Fang, S. Rampersaud, A. Hoshino, I. Matei, C. M. Kenific, M. Nakajima, A. P. Mutvei, P. Sansone, W. Buehring, H. Wang, J. P. Jimenez, L. Cohen-Gould, N. Paknejad, M. Brendel, K. Manova-Todorova, A. Magalhães, J. A. Ferreira, H. Osório, A. M. Silva, A. Massey, J. R. Cubillos-Ruiz, G. Galletti, P. Giannakakou, A. M. Cuervo, J. Blenis, R. Schwartz, M. S. Brady, H. Peinado, J. Bromberg, H. Matsui, C. A. Reis, D. Lyden, Identification of distinct nanoparticles and subsets of extracellular vesicles by asymmetric flow field-flow fractionation. *Nat. Cell Biol.* **20**, 332–343 (2018).

3. M. Kitagawa, P. Wu, R. Balkunde, P. Cunniff, D. Jackson, An RNA exosome subunit mediates cell-to-cell trafficking of a homeobox mRNA via plasmodesmata. *Science* **375**, 177–182 (2022).

4. Y. Chen, Q. Zhu, L. Cheng, Y. Wang, M. Li, Q. Yang, L. Hu, D. Lou, J. Li, X. Dong, L. P. Lee, F. Liu, Exosome detection via the ultrafast-isolation system: EXODUS. *Nat. Methods* **18**, 212–218 (2021).

5. P. Li, M. Kaslan, S. H. Lee, J. Yao, Z. Gao, Progress in exosome isolation techniques. *Theranostics* **7**, 789–804 (2017).

6. B. Shen, Y. Fang, N. Wu, S. J. Gould, Biogenesis of the posterior pole is mediated by the exosome/microvesicle protein-sorting pathway. *J. Biol. Chem.* **286**, 44162–44176 (2011).

7. K. W. Witwer, J. Wolfram, Extracellular vesicles versus synthetic nanoparticles for drug delivery. *Nat. Rev. Mater.* **6**, 103–106 (2021).

8. Y.-L. Tai, K.-C. Chen, J.-T. Hsieh, T.-L. Shen, Exosomes in cancer development and clinical applications. *Cancer Sci.* **109**, 2364–2374 (2018).

9. K. Liang, F. Liu, J. Fan, D. Sun, C. Liu, C. J. Lyon, D. W. Bernard, Y. Li, K. Yokoi, M. H. Katz,

E. J. Koay, Z. Zhao, Y. Hu, Nanoplasmonic quantification of tumor-derived extracellular vesicles in plasma microsamples for diagnosis and treatment monitoring. *Nat. Biomed. Eng.* **1**, 0021 (2017).

10. W. Song, A. C. Anselmo, L. Huang, Nanotechnology intervention of the microbiome for cancer therapy. *Nat. Nanotechnol.* **14**, 1093–1103 (2019).

11. S. Kamerkar, C. Leng, O. Burenkova, S. C. Jang, C. McCoy, K. Zhang, K. Dooley, S. Kasera, T. Zi, S. Sisó, W. Dahlberg, C. L. Sia, S. Patel, K. Schmidt, K. Economides, T. Soos, D. Burzyn, S. Sathyanarayanan, Exosome-mediated genetic reprogramming of tumor-associated macrophages by exoASO-STAT6 leads to potent monotherapy antitumor activity. *Sci. Adv.* **8**, eabj7002 (2022).

12. Y.-T. Lu, K. Delijani, A. Mecum, A. Goldkorn, Current status of liquid biopsies for the detection and management of prostate cancer. *Cancer Manag. Res.* **11**, 5271–5291 (2019).

13. R. Shah, T. Patel, J. E. Freedman, Circulating extracellular vesicles in human disease. *N. Engl. J. Med.* **379**, 958–966 (2018).

14. D. K. Sarko, C. E. McKinney, Exosomes: Origins and therapeutic potential for neurodegenerative disease. *Front. Neurosci.* **11**, 82 (2017).

15. R. C. Lai, T. S. Chen, S. K. Lim, Mesenchymal stem cell exosome: A novel stem cell-based therapy for cardiovascular disease. *Regen. Med.* **6**, 481–492 (2011).

16. M. D. Vahey, J. Voldman, High-throughput cell and particle characterization using isodielectric separation. *Anal. Chem.* **81**, 2446–2455 (2009).

17. Y. Gu, C. Chen, Z. Mao, H. Bachman, R. Becker, J. Rufo, Z. Wang, P. Zhang, J. Mai, S. Yang, J. Zhang, S. Zhao, Y. Ouyang, D. T. W. Wong, Y. Sadovsky, T. J. Huang, Acoustofluidic centrifuge for nanoparticle enrichment and separation. *Sci. Adv.* **7**, eabc0467 (2021).

18. B. H. Wunsch, J. T. Smith, S. M. Gifford, C. Wang, M. Brink, R. L. Bruce, R. H. Austin, G. Stolovitzky, Y. Astier, Nanoscale lateral displacement arrays for the separation of exosomes and

colloids down to 20 nm. *Nat. Nanotechnol.* **11,** 936–940 (2016).

19. T. Salafi, K. K. Zeming, Y. Zhang, Advancements in microfluidics for nanoparticle separation. *Lab Chip*, **17**, 11–33 (2017).

20. J. Zhou, Z. Wu, J. Hu, D. Yang, X. Chen, Q. Wang, J. Liu, M. Dou, W. Peng, Y. Wu, W. Wang, C. Xie, M. Wang, Y. Song, H. Zeng, C. Bai, High-throughput single-EV liquid biopsy: Rapid, simultaneous, and multiplexed detection of nucleic acids, proteins, and their combinations. *Sci. Adv.* **6**, eabc1204 (2020).

21. J. Zhang, C. Ji, H. Zhang, H. Shi, F. Mao, H. Qian, W. Xu, D. Wang, J. Pan, X. Fang, H. A. Santos, X. Zhang, Engineered neutrophil-derived exosome-like vesicles for targeted cancer therapy. *Sci. Adv.* **8**, eabj8207 (2022).

22. S. A. Melo, L. B. Luecke, C. Kahlert, A. F. Fernandez, S. T. Gammon, J. Kaye, V. S. LeBleu, E. A. Mittendorf, J. Weitz, N. Rahbari, C. Reissfelder, C. Pilarsky, M. F. Fraga, D. Piwnica-Worms, R. Kalluri. Glypican-1 identifies cancer exosomes and detects early pancreatic cancer. *Nature* **523**, 177–182 (2015).

23. P. Zhang, X. Zhou, M. He, Y. Shang, A. L. Tetlow, A. K. Godwin, Y. Zeng, Ultrasensitive detection of circulating exosomes with a 3D-nanopatterned microfluidic chip. *Nat. Biomed. Eng.* **3**, 438–451 (2019).

24. F. Momen-Heravi, Isolation of extracellular vesicles by ultracentrifugation, in *Extracellular Vesicles* (Humana Press, 2017), pp. 25–32.

25. K. Iwai, T. Minamisawa, K. Suga, Y. Yajima, K. Shiba. Isolation of human salivary extracellular vesicles by iodixanol density gradient ultracentrifugation and their characterizations. *J. Extracell. Vesicles* **5**, 30829 (2016).

26. A. N. Böing, E. Van Der Pol, A. E. Grootemaat, F. A. W. Coumans, A. Sturk, R. Nieuwland, Single-step isolation of extracellular vesicles by size-exclusion chromatography. *J. Extracell. Vesicles* **3**, 23430 (2014).

27. P. N. Brown, H. Yin, Polymer-based purification of extracellular vesicles, in *Extracellular Vesicles* (Humana Press, 2017), pp. 91–103.

28. R. Xu, R. J. Simpson, D. W. Greening, A protocol for isolation and proteomic characterization of distinct extracellular vesicle subtypes by sequential centrifugal ultrafiltration, in *Exosomes and Microvesicles* (Humana Press, 2017), pp. 91–116.

29. H. Wijerathne, M. A. Witek, J. M. Jackson, V. Brown, M. L. Hupert, K. Herrera, C. Kramer, A. E. Davidow, Y. Li, A. E. Baird, M. C. Murphy, S. A. Soper, Affinity enrichment of extracellular vesicles from plasma reveals mRNA changes associated with acute ischemic stroke. *Commun. Biol.* **3**, 613 (2020).

30. Y. Wan, G. Cheng, X. Liu, S. Hao, M. Nisic, C.-D. Zhu, Y.-Q. Xia, W.-Q. Li, Z.-G. Wang, W.-L. Zhang, S. J. Rice, A. Sebastian, I. Albert, C. P. Belani, S.-P. Zheng, Rapid magnetic isolation of extracellular vesicles via lipid-based nanoprobes. *Nat. Biomed. Eng.* **1**, 0058 (2017).

31. Y. Kim, X. Zhao, Magnetic soft materials and robots. *Chem. Rev.* **122**, 5317–5364 (2022).

32. J. Z. Nordin, Y. Lee, P. Vader, I. Mäger, H. J. Hohansson, W. Heusermann, O. P. B. Wiklander, M. Hällbrink, Y. Seow, J. J. Bultema, J. Gilthorpe, T. Davies, P. J. Faorchild, S. Gabrielsson, N. C. Meisner-Kober, J. Lehtiö, C. I. E. Smith, M. J. A. Wood, S. E. L. Andaloussi, Ultrafiltration with size-exclusion liquid chromatography for high yield isolation of extracellular vesicles preserving intact biophysical and functional properties. *Nanomedicine* **11**, 879–883 (2015).

33. M. J. Skaug, C. Schwemmer, S. Fringes, C. D. Rawlings, A. W. Knoll. Nanofluidic rocking Brownian motors. *Science* **359**, 1505–1508 (2018).

34. J. Y. Kim, S. W. Ahn, S. S. Lee, J. M. Kim, Lateral migration and focusing of colloidal particles and DNA molecules under viscoelastic flow. *Lab Chip* **12**, 2807–2814 (2012).

35. J. Reboud, Y. Bourquin, R. Wilson, G. S. Pall, M. Jiwaji, A. R. Pitt, A. Graham, A. P. Waters, J. M. Cooper, Shaping acoustic fields as a toolset for microfluidic manipulations in diagnostic technologies. *Proc. Natl. Acad. Sci. U.S.A.* **109**, 15162–15167 (2012).

36. D. J. Collins, R. O'Rorke, C. Devendran, Z. Ma, J. Han, A. Neild, Y. Ai, Self-aligned acoustofluidic particle focusing and patterning in microfluidic channels from channel-based acoustic waveguides. *Phys. Rev. Lett.* **120**, 1–6 (2018).

37. C. Devendran, D. J. Collins, Y. Ai, A. Neild, Huygens-Fresnel acoustic interference and the development of robust time-averaged patterns from traveling surface acoustic waves. *Phys. Rev. Lett.* **118**, 154501 (2017).

38. C. Devendran, K. Choi, J. Han, Y. Ai, A. Neild, D. J. Collins, Diffraction-based acoustic manipulation in microchannels enables continuous particle and bacteria focusing. *Lab Chip* **20**, 2674–2688 (2020).

39. J. Friend, L. Y. Yeo, Microscale acoustofluidics: Microfluidics driven via acoustics and ultrasonics. *Rev. Mod. Phys.*, **83**, 647–704 (2011).

40. M. Wiklund, C. Günther, R. Lemor, M. Jäger, G. Fuhr, H. M. Hertz, Ultrasonic standing wave manipulation technology integrated into a dielectrophoretic chip. *Lab Chip* **6**, 1537–1544 (2006).

41. Y. Deng, W. A. Benalcazar, Z. G. Chen, M. Oudich, G. Ma, Y. Jing, Observation of degenerate zero-energy topological states at disclinations in an acoustic lattice. *Phys. Rev. Lett.* **128**, 174301 (2022).

42. Y. Li, X. Jiang, B. Liang, J. C. Cheng, L. Zhang, Metascreen-based acoustic passive phased array. *Phys. Rev. Appl.* **4**, 024003 (2015).

43. R. Tao, S. A. Hasan, H. Z. Wang, J. Zhou, J. T. Luo, G. McHale, D. Gibson, P. Canyelles-Pericas, M. D. Cooke, D. Wood, Y. Liu, Q. Wu, W. P. Ng, T. Franke, Y. Q. Fu, Bimorph material/structure designs for high sensitivity flexible surface acoustic wave temperature sensors. *Sci. Rep.* **8**, 9052 (2018).

44. H. G. Kraus, Huygens–Fresnel–Kirchhoff wave-front diffraction formulation: Spherical waves, *J. Opt. Soc. Am. A* **6**, 1196–1205 (1989).

45. A. S. Dukhin, P. J. Goetz, Chapter 3—Fundamentals of acoustics in homogeneous liquids:

Longitudinal rheology. *Stud. Interface Sci.* **24**, 91–125 (2010).

46. G. Lajoinie, T. Segers, M. Versluis, High-frequency acoustic droplet vaporization is initiated by resonance. *Phys. Rev. Lett.* **126**, 034501 (2021).

47. K. Melde, A. G. Mark, T. Qiu, P. Fischer, Holograms for acoustics. *Nature* **537**, 518–522 (2016).

48. M. Baudoin, J. C. Gerbedoen, A. Riaud, O. B. Matar, N. Smagin, J. L. Thomas, Folding a focalized acoustical vortex on a flat holographic transducer: Miniaturized selective acoustical tweezers. *Sci. Adv.* **5**, eaav1967 (2019).

49. X. Ding, P. Li, S. S. Lin, Z. S. Stratton, N. Nama, F. Guo, D. Slotcavage, X. Mao, J. Shi, F. Costanzoa, T. J. Huang. Surface acoustic wave microfluidics. *Lab Chip* **13**, 3626–3649 (2013).

50. C. R. Courtney, C.-K. Ong, B. W. Drinkwater, P. D. Wilcox, C. Demore, S. Cochran, P Glynne-Jones, M. Hill, Manipulation of microparticles using phase-controllable ultrasonic standing waves. *J. Acoust. Soc. Am.* **128**, EL195–EL199 (2010).

51. A. Marzo, B. W. Drinkwater, Holographic acoustic tweezers. *Proc. Natl. Acad. Sci.* **116**, 84–89 (2019).

52. D. Ahmed, T. Baasch, N. Blondel, N. Läubli, J. Dual, B. J. Nelson, Neutrophil-inspired propulsion in a combined acoustic and magnetic field. *Nat. Commun.* **8**, 770 (2017).

53. N. Fang, D. Xi, J. Xu, M. Ambati, W. Srituravanich, C. Sun, X. Zhang, Ultrasonic metamaterials with negative modulus, *Nat. Mater.* **5**, 452–456 (2006).

54. M. W. H. Kirkness, C. S. Korosec, N. R. Forde, Modified pluronic F127 surface for bioconjugation and blocking nonspecific adsorption of microspheres and biomacromolecules. *Langmuir* **34**, 13550–13557 (2018).

55. B. Raeymaekers, C. Pantea, D. N. Sinha, Manipulation of diamond nanoparticles using bulk acoustic waves. *J. Appl. Phys.* **109**, 014317 (2011).

56. R. Barnkob, P. Augustsson, T. Laurell, H. Bruus, Acoustic radiation-and streaming-induced microparticle velocities determined by microparticle image velocimetry in an ultrasound symmetry plane. *Phys. Rev. E* **86**, 056307 (2012).

57. H. Li, J. R. Friend, L. Y. Yeo, Surface acoustic wave concentration of particle and bioparticle suspensions. *Biomed. Microdevices* **9**, 647–656 (2007).

58. N. Zhang, A. Horesh, C. Floer, J. Friend, Unapodization: A method to produce laterally uniform surface acoustic waves for acoustofluidics, *J. Micromech. Microeng.* **31**, 104001 (2021).

59. N. Zhang, A. Horesh, Q. Manor, J. Friend, Powerful acoustogeometric streaming from dynamic geometric nonlinearity. *Phys. Rev. Lett.* **126**, 164502 (2021).

60. J. Rufo, F. Cai, J. Friend, M. Wiklund, T. J. Huang, Acoustofluidics for biomedical applications, *Nat. Rev. Methods Primers* **2**, 30 (2022).

61. A. Ozcelik, J. Rufo, F. Guo, Y. Gu, P. Li, J. Lata, T. J. Huang, Acoustic tweezers for the life sciences. *Nat. Methods* **15**, 1021–1028 (2018).

62. P. Zhang, H. Bachman, A. Ozcelik, T. J. Huang, Acoustic microfluidics. *Annu. Rev. Anal. Chem.* **13**, 17–43 (2020).

63. J. Rufo, P. Zhang, R. Zhong, L. P. Lee, T. J. Huang, A sound approach to advancing healthcare systems: The future of biomedical acoustics. *Nat. Commun.* **13**, 3459 (2022).

64. S. Yang, Z. Tian, Z. Wang, J. Rufo, P. Li, J. Mai, J. Xia, H. Bachman, P.-H. Huang, M. Wu, C. Chen, L. P. Lee, T. J. Huang, Harmonic acoustics for dynamic and selective particle manipulation. *Nat. Mater.* **21**, 540–546 (2022).

65. P. Liu, Z. Tian, K. Yang, T. D. Naquin, N. Hao, H. Huang, J. Chen, Q. Ma, H. Bachman, P. Zhang, X. Xu, J. Hu, T. J. Huang, Acoustofluidic black holes for multifunctional in-droplet particle manipulation. *Sci. Adv.* **8**, eabm2592 (2022).

66. J. C. Ndukaife, A. V. Kildishev, A. Nnanna, V. M. Shalaev, S. T. Wereley, A. Boltasseva,

Long-range and rapid transport of individual nano-objects by a hybrid electrothermoplasmonic nanotweezer. *Nat. Nanotechnol.* **11**, 53–59 (2016).

67. P. Zhang, J. Rufo, C. Chen, J. Xia, Z. Tian, L. Zhang, N. Hao, Z. Zhong, Y. Gu, K. Chakrabarty, T. J. Huang, Acoustoelectronic nanotweezers enable dynamic and large-scale control of nanomaterials. *Nat. Commun.* **12**, 3844 (2021).

68. D. N. Basov, M. M. Fogler, F. J. García de Abajo, Polaritons in van der Waals materials. *Science* **354**, aag1992 (2016).

69. M. M. Kim, A. L. Zydney, Effect of electrostatic, hydrodynamic, and Brownian forces on particle trajectories and sieving in normal flow filtration. *J. Colloid Interface Sci.* **269**, 425–431 (2004).

70. P. B. Muller, R. Barnkob, M. J. H. Jensen, H. Bruus, A numerical study of microparticle acoustophoresis driven by acoustic radiation forces and streaming-induced drag forces. *Lab Chip* **12**, 4617–4627 (2012).

71. F. Guo, Z. Mao, Y. Chen, Z. Xie, J. P. Lata, P. Li, L. Ren, J. Liu, J. Yang, M. Dao, S. Suresh, T. J. Huang, Three-dimensional manipulation of single cells using surface acoustic waves. *Proc. Natl. Acad. Sci. U.S.A.* **113**, 1522–1527 (2016).
